# Structural similarities between SARS-CoV2 3CL^pro^ and other viral proteases suggest potential lead molecules for developing broad spectrum antivirals

**DOI:** 10.3389/fchem.2022.948553

**Published:** 2022-10-06

**Authors:** Khushboo Bafna, Christopher L. Cioffi, Robert M. Krug, Gaetano T. Montelione

**Affiliations:** ^1^ Department of Chemistry and Chemical Biology, Rensselaer Polytechnic Institute, Troy, NY, United States; ^2^ Center for Biotechnology and Interdisciplinary Sciences, Rensselaer Polytechnic Institute, Troy, NY, United States; ^3^ Department of Molecular Biosciences, John Ring LaMontagne Center for Infectious Disease, Institute for Cellular and Molecular Biology, University of Texas at Austin, Austin, TX, United States

**Keywords:** COVID-19, drug discovery, structural bioinformatics, virtual docking, structure based dendrograms, topology diagrams, viral protease inhibitors, 3CL^pro^ (Mpro)

## Abstract

Considering the significant impact of the recent COVID-19 outbreak, development of broad-spectrum antivirals is a high priority goal to prevent future global pandemics. Antiviral development processes generally emphasize targeting a specific protein from a particular virus. However, some antiviral agents developed for specific viral protein targets may exhibit broad spectrum antiviral activity, or at least provide useful lead molecules for broad spectrum drug development. There is significant potential for repurposing a wide range of existing viral protease inhibitors to inhibit the SARS-CoV2 3C-like protease (3CL^pro^). If effective even as relatively weak inhibitors of 3CL^pro^, these molecules can provide a diverse and novel set of scaffolds for new drug discovery campaigns. In this study, we compared the sequence- and structure-based similarity of SARS-CoV2 3CL^pro^ with proteases from other viruses, and identified 22 proteases with similar active-site structures. This structural similarity, characterized by secondary-structure topology diagrams, is evolutionarily divergent within taxonomically related viruses, but appears to result from evolutionary convergence of protease enzymes between virus families. Inhibitors of these proteases that are structurally similar to the SARS-CoV2 3CL^pro^ protease were identified and assessed as potential inhibitors of SARS-CoV2 3CL^pro^ protease by virtual docking. Several of these molecules have docking scores that are significantly better than known SARS-CoV2 3CL^pro^ inhibitors, suggesting that these molecules are also potential inhibitors of the SARS-CoV2 3CL^pro^ protease. Some have been previously reported to inhibit SARS-CoV2 3CL^pro^. The results also suggest that established inhibitors of SARS-CoV2 3CL^pro^ may be considered as potential inhibitors of other viral 3C-like proteases.

## Introduction

Coronaviruses (CoVs) cause human respiratory diseases. While several human coronaviruses cause relatively mild respiratory infections, three coronaviruses cause severe respiratory diseases in humans: Severe Acute Respiratory Syndrome (SARS), Middle East Respiratory Syndrome (MERS), and Corona Virus Infectious Disease 2019 (COVID-19) (de Wit et al., 2016; Wu et al., 2020; Zhou et al., 2020). The current COVID-19 pandemic has had a devastating impact on public health and global economies. The etiologic cause of COVID-19 disease is the novel SARS-CoV2 virus (Wu et al., 2020; Zhou et al., 2020). While both vaccines and approved antiviral drugs (Mei and Tan, 2021; Burki, 2022) are now available, immuno- and antiviral-resistant viral variants continue to emerge, with severe ongoing public health consequences. Considering the high mutation rate of SARS-CoV2 (McLean et al., 2022), an important focus of current research is the development of therapeutic strategies and molecules that address and suppress antiviral resistance.

Coronaviruses, including SARS-CoV2, are enveloped positive-strand RNA viruses. Their genome comprises a single, large (27-34 kilobase) single-stranded RNA, which is directly translated by host cells. The SARS-CoV2 genome encodes 4 structural proteins, 16 non-structural proteins (NSPs) which carry out crucial intracellular functions, and 9 accessory proteins (Gordon et al., 2020; Wu et al., 2020). Many of these proteins, and their host binding partners (Gordon et al., 2020), are potential targets for development of antiviral therapeutics for COVID-19. Translation of the viral RNA results in the synthesis of two polyproteins that are processed by two virally-encoded cysteine proteases, the papain-like protease (PL^pro^), a part of Non-Structural Protein 3 (NSP3), and a 3C-like protease (3CL^pro^), which is also referred to as Non-Structural Protein 5 (NSP5), or as the main protease (M^pro^). Both PL^pro^ and 3CL^pro^ proteases are required for virus replication and are targets for antiviral development.

Considering the urgency for identifying effective antiviral drugs for COVID-19, and the usually lengthy process involved in approving candidate drugs for safe human use, an important approach has been to identify existing drugs and inhibitors that can be optimized as potent and safe antivirals. Viral proteases have been successfully targeted for the development of antiviral drugs against human immunodeficiency virus-1 (HIV-1), hepatitis C virus (HCV) (Wlodawer and Vondrasek, 1998; Kwo and Vinayek, 2011; McGivern et al., 2015; Ghosh et al., 2016), and most recently for SARS-CoV2 (Beck et al., 2020; Nguyen et al., 2020; Boras et al., 2021; Dampalla et al., 2021; Liu et al., 2022; Narayanan et al., 2022). Here we outline the potential of using existing inhibitors directed to other viral proteases as lead molecules for developing new drugs targeting the SARS-CoV2 3CL^pro^ protease.

Work over the past ∼15 years on the SARS-CoV 3CL protease has provided an extensive understanding of structure-activity relationships of lead molecules suitable for drug discovery efforts (Anand et al., 2003; Yang et al., 2003; Yang et al., 2006; Akaji et al., 2011; Hilgenfeld and Peiris, 2013; Pillaiyar et al., 2016; Gordon et al., 2020). Although these drug development efforts have been focused on specific proteases, in some cases broad spectrum activities have been documented. We define broad spectrum protease inhibitors as molecules that effectively inhibit proteases from viral strain variants, or even proteases from different viral species. Particularly noteworthy are several hepatitis C virus (HCV) drugs developed as inhibitors of the HCV NS3/4A protease, which also have activity as micromolar inhibitors of SARS-CoV2 virus replication in cell culture (Bafna et al., 2020; Bafna et al., 2021; Gammeltoft et al., 2021; Lo et al., 2021). Another example, with a narrower target range, nirmatrelvir, a peptidomimetic developed as an inhibitor of the SARS-CoV2 virus and a key component of the Pfizer antiviral drug combination Paxlovid™, has good activity as an inhibitor of 3CL^pro^ from a wide range of SARS-CoV2 viral strains (Ullrich et al., 2022). Rupintrivir also has activity against a broad range of 3CL^pro^—type viral proteases from corona viruses, coxsackie viruses, rhinoviruses, and entroviruses (Lockbaum et al., 2021). Broad spectrum antiviral activity may be important for development of drugs that can suppress the evolution of viral resistance.

While in most cases broad spectrum activity of 3CL^pro^ inhibitors has been assessed by experimental screening using protease inhibition or antiviral activity assays, some success has also been achieved by using rational approaches and virtual screening. For example, several HCV protease inhibitor drugs were initially proposed as inhibitors of SARS-CoV2 3CL^pro^ based on structural bioinformatics studies which identified structural similarity in and around the active sites of these two proteases (Bafna et al., 2020). This hypothesis was subsequently validated by virtual docking studies, and experimental biochemical protease inhibition and cell-based viral inhibition assays (Bafna et al., 2021; Gammeltoft et al., 2021).

In this study, we expand our earlier structural bioinformatics analysis to identify more than 20 proteases from a wide range of positive single-stranded RNA viruses for which the 3D structures of the binding-site cleft is similar to SARS-CoV2 3CL^pro^. These viral proteases belong to the well-recognized PA superfamily of chymotrypsin-like proteases (Bazan and Fletterick, 1988; Gorbalenya et al., 1989; Kanitz et al., 2019), which includes proteases from species across the tree of life (Laskar et al., 2012; Monttinen et al., 2019). Phylogenetic and structural topology analysis indicates that the proteins from these various viral protease clades have evolutionarily converged on similar active site structures. For many of these proteases, medicinal chemistry efforts have previously identified inhibitor molecules. Our virtual docking experiments suggest that many of these known protease inhibitors have potential as lead molecules for developing novel drugs directed to SARS-CoV2 3CL^pro^. In a few cases, these inhibitors developed for these other viral proteases have already been shown to inhibit of SARS-CoV2 3CL^pro^ and/or viral replication in cell-based assays at micromolar concentrations.

## Computational methods

### Structural bioinformatics

Proteins that are structurally similar to SARS-CoV2 3CL^pro^ were identified using the DALI (Distance matrix ALIgnment) (Holm and Sander, 1995; Holm, 2020a) server (http://ekhidna2.biocenter.helsinki.fi). The first two domains of the SARS-CoV2 3CL^pro^ were used as a structural template to search for structurally-similar viral proteases in the PDB25 database (Holm, 2020b). PDB25 is a non-redundant subset of the PDB, consisting of representative structures from clades clustered at 25% sequence identity. In addition, the all-against-all structure comparison option available on the DALI server was used to generate structure-based dendrograms of these viral proteases. Sequence based phylogenetic trees were generated using Clustal Omega (Madeira et al., 2022) available on the European Bioinformatics (EBI) website (https://www.ebi.ac.uk/Tools/msa/clustalo/). Clustal Omega uses the HHalign (Soding, 2005) algorithm with the Gonnet (Gonnet et al., 1992) transition matrix. Sequence information for each protein listed in Table 1 was obtained in FASTA format from the respective PDB entry.

**TABLE 1:** Viral proteases identified from DALI search.

PDB id	Z score	RMSD	Protein name^a^ (Cys/Ser protease)	Organism
4WME	33.6	0.9	3C-Like protease (Cys)	Middle East respiratory syndrome (MERS) related coronavirus
6JIJ	34.3	0.8	Main protease (Cys)	Murine hepatitis virus (MHV) strain A59
4ZUH	33.9	0.9	3C-Like protease (Cys)	Porcine epidemic diarrhea virus (PEDV)
2Q6F	33.3	1.2	Main protease (Cys)	Infectious bronchitis virus (IBV)
5LAK	14.3	3.1	3C-Like protease (Cys)	Cavally virus (CV)
1LVM	14.0	3.1	3C-Like protease (Cys)	Tobacco etch virus (TEV)
3ZZ9	12.4	2.8	3C-Like protease (Cys)	Coxsackievirus (CAV) B3
5FX6	12.4	2.7	3C-Like protease (Cys)	Rhinovirus (RHV)
3Q3Y	12.4	2.8	3C-Like protease (Cys)	Human enterovirus (HEV) 93
2H9H	12.3	3.2	3C- proteinase (Cys)	Hepatitis A virus (HAV)
1MBM	12.2	2.7	NSP4 proteinase (Ser)	Equine arteritis virus (EAV)
5BPE	12.3	2.8	3C Protease (Cys)	Human enterovirus (HEV) A71
5Y4L	11.8	2.8	3C-Like protease (NSP4) (Ser)	Porcine reproductive and respiratory syndrome virus (PRRSV)
4INH	11.6	3.0	Protease (Cys)	Norwalk virus (NWV)
5E0H	11.6	2.9	3C-Like protease (Cys)	Norovirus (NOV)
4ASH	11.3	2.8	NS6 protease (Cys)	Murine norovirus 1 (MNOV)
6L0T	10.5	3.3	3C Protease (Cys)	Senecavirus A (SNV)
2WV4	10.7	3.0	3C Protease (Cys)	Foot-and-mouth disease virus (FMDV)
3L6P	9.0	2.8	NS2B/NS3 protease (Ser)	Dengue virus (DENV)
2GGV	9.0	2.8	NS2B/NS3 protease (Ser)	West Nile virus (WNV)
5LC0	8.5	2.9	NS2B/NS3 protease (Ser)	Zika virus (ZKV)
2P59	8.1	3.0	NS3/4A protease (Ser)	Hepatitis C virus (HCV)

a Based on their structures, we consider all of these proteases as 3C-Like proteases; the name provided is a common name reported in the literature.

### Virtual docking

Virtual docking was done using the open source *Autodock* suite (Morris et al., 2009). *AutoDockTools* was used for coordinate preparation, docking, and analysis of results, as described previously (Bafna et al., 2021). SARS-CoV2 3CL^pro^ atomic coordinates were obtained from X-ray crystal structure PDB id 6Y2G (Zhang et al., 2020), and structural water molecules were removed. Three-dimensional coordinates for ligand molecules were obtained from the PDB (http://www.rcsb.org/) or from chemical structure databases ChemSpider (http://www.chemspider.com/) and DrugBank (https://www.drugbank.ca/). Docking calculations were carried out using a cpu cluster at the Rensselaer Polytechnic Institute Center for Computer Innovations (CCI) (https://cci.rpi.edu/). Atomic coordinates for best-scoring conformation obtained in each docking simulation, for each drug-protein complex, were saved in PDB format for analysis. These protein - ligand complexes were analyzed in detail using the open source *PyMol* molecular visualization tool (DeLano, 2009) and fully automated *Protein-Ligand Interaction Profiler* (Salentin et al., 2015) (https://projects.biotec.tu-dresden.de/plip-web/plip).

## Results

### Structural analogs of SARS-CoV2 3CL^pro^

**FIGURE 1:**
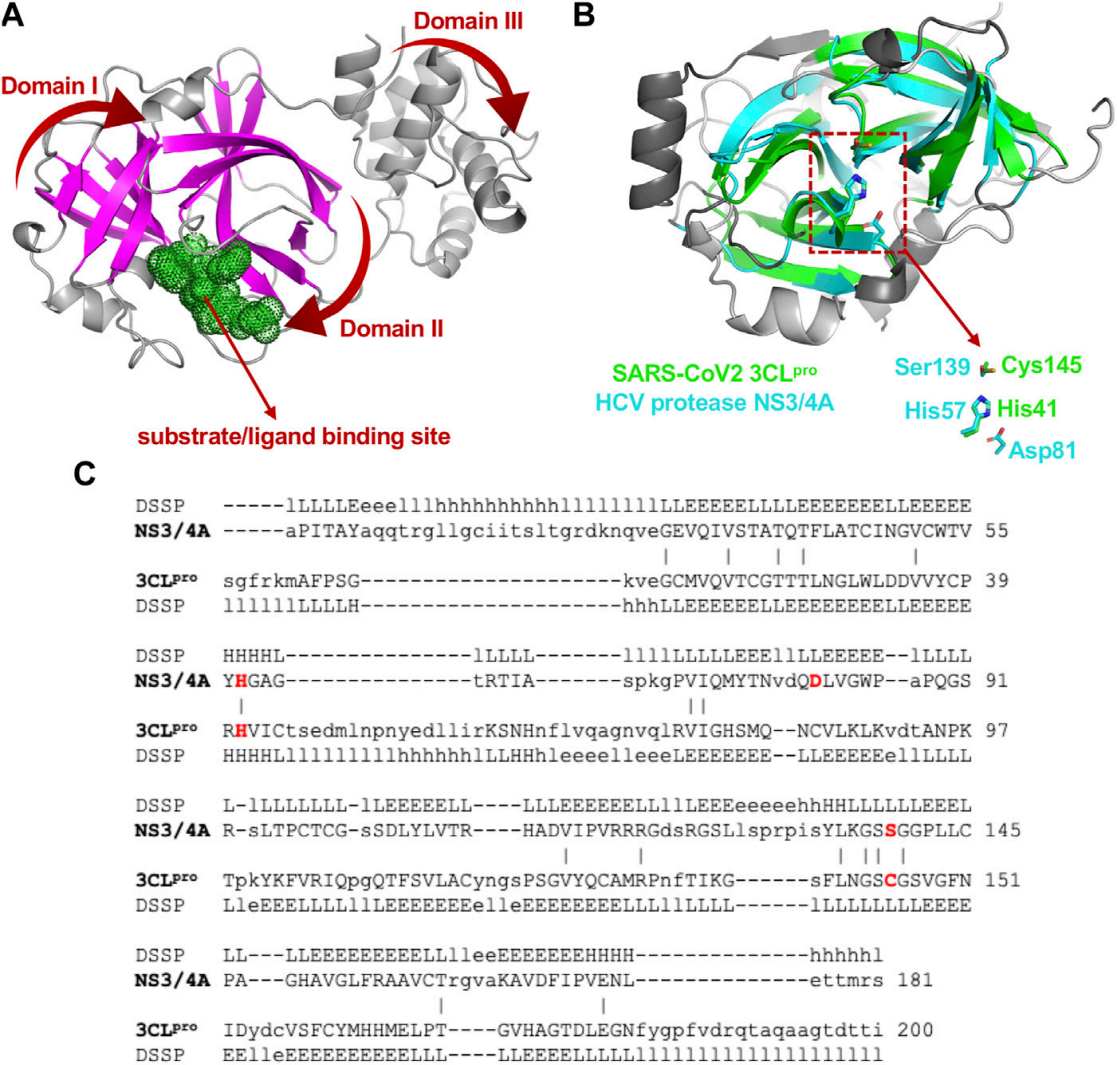
Structural superimposition and structure-based sequence alignments of SARS-CoV2 3CL^pro^ and HCV NS3/4A proteases. **(A)** Three-dimensional structures of SARS-CoV2 3CL^pro^. The β-strands forming the characteristic β-barrels are colored in magenta. Other secondary structure elements are shown as cartoon representation colored in gray. **(B)** The backbone structure of the SARS-CoV2 3CL^pro^, PDB 6Y2G (green) is superimposed on the backbone structure of hepatitis C virus protease HCV NS3/4A, PDB 2P59 (cyan). The regions identified by DALI server as structurally-analogous are shown in color (green and cyan), and the regions that are not structurally-analogous are shown in gray. This superimposition of backbone atoms results in superimposition of the catalytic residues Cys145 and His41 of the SARS-CoV2 3CL^pro^ with Ser139 and His57 of HCV NS3/4A protease. Residue Asp81 of the HCV protease catalytic triad is also shown. **(C)** Structure-based sequence alignment of HCV NS3/4A and SARS-CoV2 3CL^pro^. Catalytic residues of HCV NS3/4A (His57, Asp81 and Ser139) and SARS-Cov2 3CL^pro^ (His41 and Cys145) are highlighted in bold red. Three-state secondary structure definitions (H = helix, E = sheet, L = coil) are shown for each amino acid sequence. Structurally equivalent residues are in uppercase, structurally non-equivalent residues (e.g. in loops) are in lowercase. Identical amino acids are marked by vertical bars. Adapted from Bafna et al., 2021.

3CL^pro^ of SARS-CoV2 is a 67.6 kDa homodimeric cysteine protease. It has about 97% sequence identity with the corresponding 3CL^pro^ of the SARS-CoV virus responsible for the 2003 SARS pandemic. Not surprisingly, the 1.75 Å X-ray crystal structure of SARS-CoV2 3CL^pro^ protease (Jin et al., 2020; Zhang et al., 2020) demonstrates its structure is very similar to this SARS-CoV 3CL^pro^ protease (Anand et al., 2003; Yang et al., 2003). Both of these proteases contain three domains. Domains I and II adopt a double β-barrel fold, with the substrate binding site located in a shallow cleft between two antiparallel β-barrels (Figure 1A). Both of these 3CL^pro^ proteases also have an additional C terminal helical-bundle domain, Domain III (also shown in Figure 1A), which stabilizes their homodimer forms (Shi and Song, 2006; Nashed et al., 2022).

The 3D structure of Domains I and II of SARS-CoV2 3CL^pro^, including the double β-barrel fold and the substrate binding cleft, was used as input for searching for structurally-similar proteins in the PDB25 database using the DALI server. The DALI server compares superimposition-independent distance matrices, accounting for gaps, insertions, and rearrangements, to define a structural superimposition and a structure-based sequence alignment (Holm and Sander, 1993). Structural similarity is reported as Z-score, relative to the distribution of all-vs-all pair-wise structural similarity scores in the queried structural database. A higher Z-score means the structures have higher structural similarity in their ordered regions (Holm and Sander, 1995).

The fold architectures of Domains I and II of CoV 3CL^pro^ proteases are well known to be similar to those of chymotrypsin-like proteases and the 3C family of viral proteases (Anand et al., 2002; Monttinen et al., 2019). Using domains I and II of the SARS-CoV2 3CL^pro^ as a query, our DALI search of the PDB identified several 3C-like proteases, including the HCV NS3/4A protease, as structurally-similar (Bafna et al., 2020; Bafna et al., 2021). These SARS-CoV2 3CL^pro^ and HCV NS3/4A protease structures have a structural similarity Z score = +8.1, and overall backbone root-mean-squared deviation for structurally-similar regions of ∼ 3.0 Å. Like all 3C-like proteases, the HCV NS3/4A protease has a double β-barrel fold, with relative domain orientations similar to those of the SARS-CoV and SARS-CoV2 3CL^pro^ proteases, with a substrate binding site located in a shallow cleft between its two six-to eight-stranded antiparallel β-barrels. Superimposition of the backbone structures of these two proteases results also in superimposition of their active-site catalytic residues, His41/Cys145 and His57/Ser139 of SARS-CoV2 3CL^pro^ and HCV NS3/4A proteases, respectively, with remarkable structural similarity in the substrate binding cleft (Figure 1B), despite very little sequence identity in the pair-wise structure-based sequence alignment (Figure 1C). Our observation of this structural similarity between SARS-CoV2 3CL^pro^ and HCV proteases led us to studies of known HCV NS3/4A protease inhibitors as inhibitors of SARS-CoV2 3CL^pro^ enzyme activity and virus replication (Bafna et al., 2021).

The DALI analysis identified 22 additional viral proteins (Table 1) to which SARS-CoV2 3CL^pro^ is more structurally-similar than it is to HCV NS3/4A protease. Although many other structurally-similar proteases across the PA superfamily (Monttinen et al., 2019) were also identified, in this analysis we focused on structural-similarity between the 3CL proteases of positive single-strand RNA viruses belonging to the virus Kingdom *Orthornavirae* (RNA viruses), and in the Phyla *Pisuviricota* and *Kitrinoviricota* which include eukaryotic viruses. Many of the proteins reported in Table 1 are 3C-like proteases from important virus pathogens, including human hepatitis A, dengue, coxsackie, Norwalk, entro-, foot-and-mouth disease, West Nile, and Zika viruses. The DALI structural similarity Z scores, using domains I and II of SARS-CoV2 3CL^pro^ as a search template, on each of the proteases listed in Table 1 are all higher than (more structurally similar) the Z score to HCV NS3/4A protease; i.e. these Z scores are all > + 8. As some inhibitors of HCV NS3/4A protease are now known to both inhibit SARS-CoV2 3CL^pro^ enzyme activity and to suppress the SARS-CoV2 virus replication in cell culture at 1–50 μM concentrations (Bafna et al., 2021; Gammeltoft et al., 2021), these simple bioinformatics search results suggest a significant potential for repurposing the known inhibitors of these various proteases for treating COVID-19, as well as for using them as lead molecules for structure-based drug design efforts focused on developing novel inhibitors of SARS-CoV2 3CL^pro^. These bioinformatics results also suggest the converse; using SARS-CoV2 3CL^pro^ inhibitors as lead molecules for developing drugs targeted to 3C-like proteases of these other viruses.

**FIGURE 2:**
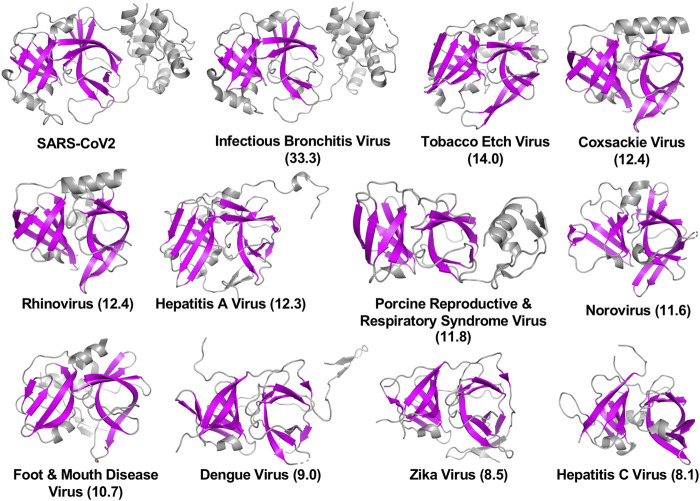
Three-dimensional structures of viral proteases that have the double β-barrel fold like the SARS-CoV2 3CL^pro^. The β-strands forming the characteristic β-barrels are colored in magenta. Other secondary structure elements are shown as cartoon representation colored in gray. Dali Z scores to SARS-CoV2 3CL^pro^ are shown in parentheses.

The viral proteases identified as structurally similar to SARS-CoV2 3CL^pro^ contain variations on the characteristic double β-barrel two-domain architecture (Figure 1A), with active sites located at the interface between the two domains. The 3D structures of some of representative viral proteases in Table 1, each having double β-barrel architectures similar to SARS-CoV2 3CL^pro^ domains I and II, are illustrated in Figure 2. The β-strands of the double β-barrel architecture, formed by 6–7 β-strands, respectively, are colored in magenta while the rest of these 3D structures (i.e., alpha helices and loops) are colored gray. The structure of one protomer of the dimeric SARS-CoV2 3CL^pro^ is also shown for comparison. These remarkable overall structural similarities across proteins from a wide taxonomic range of viral families supports the potential of developing broad spectrum inhibitors useful as lead molecules for developing new drugs targeting several viral 3CL proteases.

### Viral taxonomy

**FIGURE 3:**
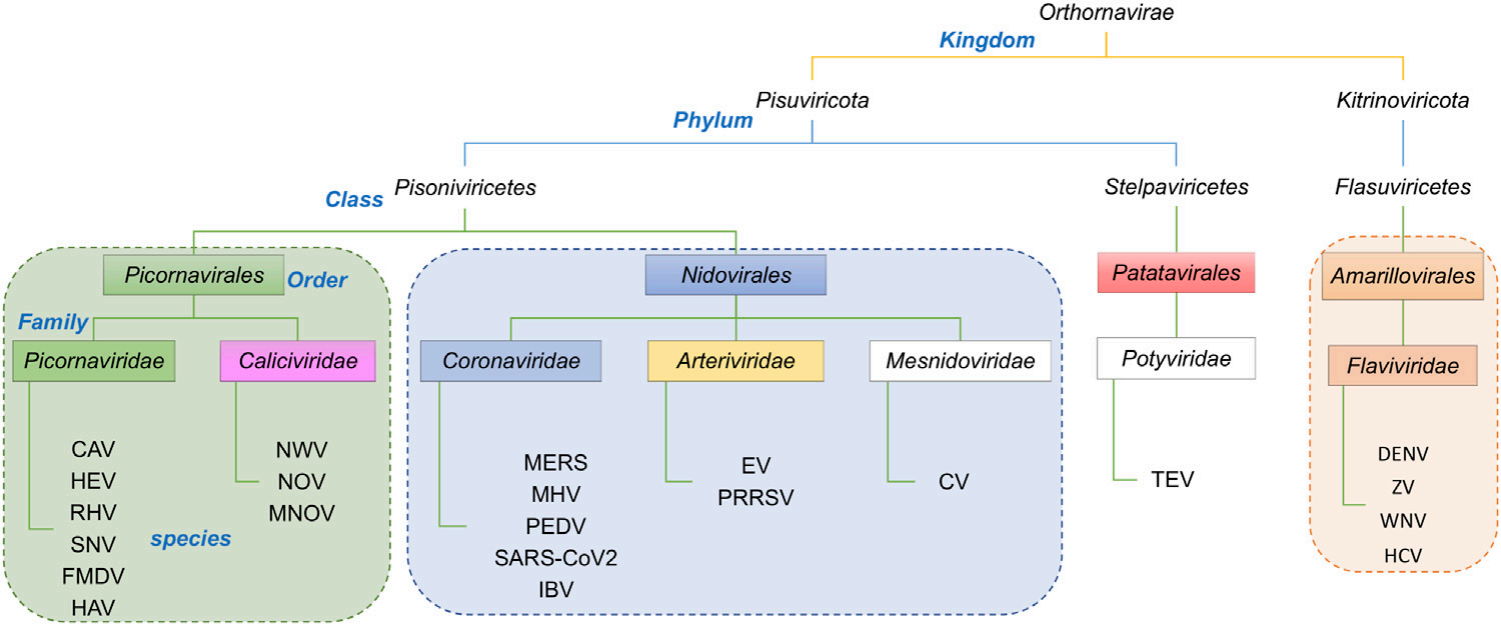
Taxonomic lineage of positive strand RNA viruses listed in Table 1. These viruses belong to two phyla in the Kingdom *Orthornavirae*. This evolutionary information is obtained from https://www.ncbi.nlm.nih.gov/Taxonomy/Browser/wwwtax.cgi.

The taxonomic lineages of the viruses associated with the proteins in Table 1 are summarized in Figure 3. They belong to two Phyla, *Pisuviricota* and *Kitrinoviricota*. Phylum *Pisuviricota* includes Classes (and Orders): *Pisoniviricetes* (Orders *Picornavirales* and *Nidovirales*), and *Stelpaviricetes* (Order *Patatavirales*). The *Picornavirales* viruses include the Families *Picornaviridae* [e.g., human hepatitis A virus (HAV)] and *Caliciviridae* [e.g., human Norwalk virus (NWV)]. The Order *Nidovirales* viruses includes the Families (or subfamilies) *Coronaviridae* [e.g., human SARS coronaviruses (SARS-CoV)], *Arteriviridae* [e.g., Equine Arteritis Virus (EAV)], and *Mesnidoviridae* [e.g., Cavally virus (CV)]. Tobacco etch mosaic virus (TEV), a common biotechnology reagent, belongs to the Class *Stelpaviricetes*, Order *Patatavirales*, of the Phylum *Pisuviricota*. The second phylum represented in the viral proteases returned by the DALI search (Table 1), *Kitrinoviricota*, includes the Family *Flaviviridae* of Class *Flasuviricetes*, Order *Amarillovirales*. The viruses of this family include flaviviruses [e.g., human dengue virus (DENV)], and hepaciviruses [e.g., human hepatitis C virus (HCV)].

### Structure- and sequence-based dendrograms

**FIGURE 4:**
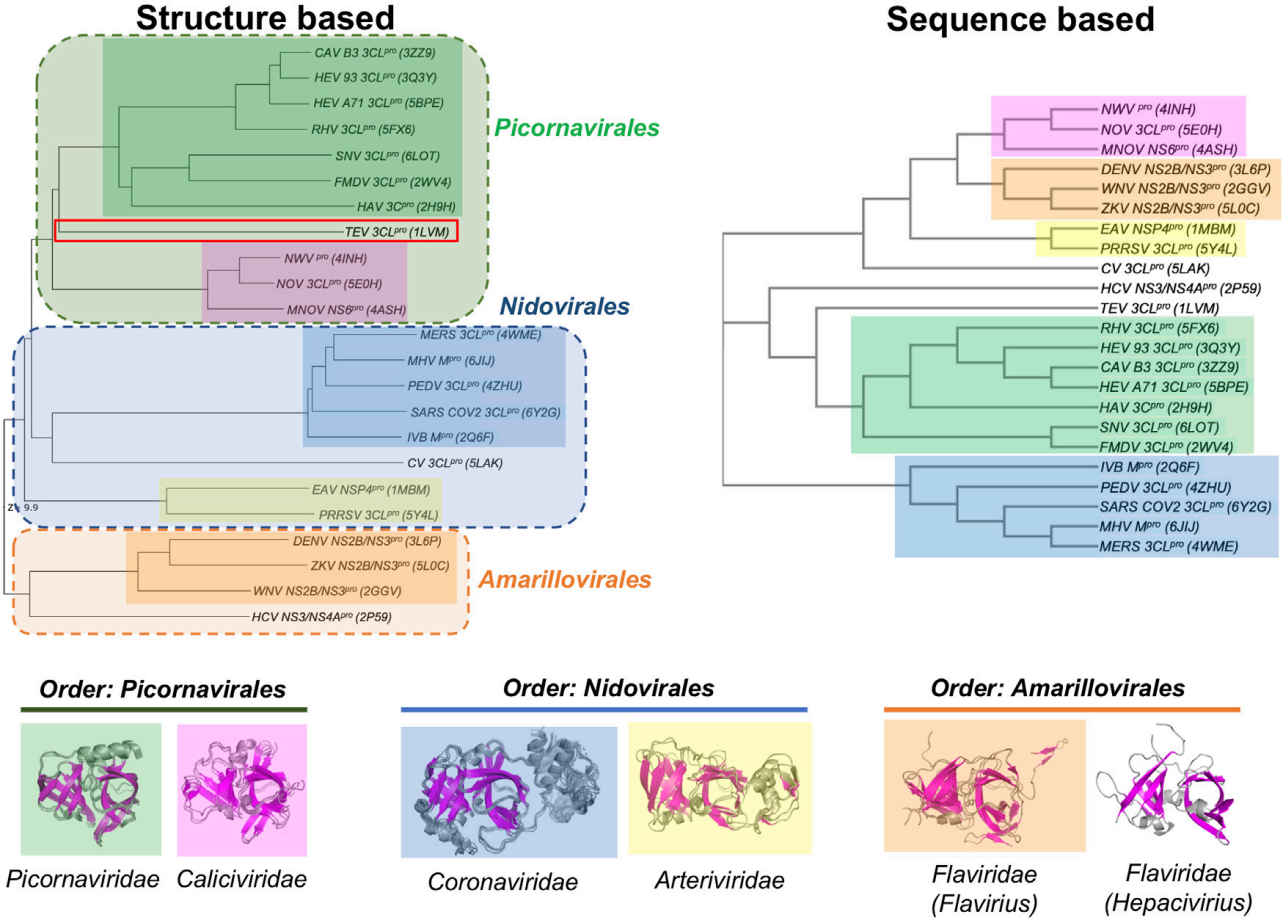
Dendrograms showing the structure-based vs. sequence-based relationships of viral proteases. In both the structure-based and sequence-based dendrograms, proteases belonging to the members within the same viral Family cluster together in clades, represented by different colored boxes. Superimpositions of the corresponding structures in each Family clade is shown below the dendrograms.

Despite these similarities in their double β-barrel architecture (Figure 2), there are also striking differences in the overall structures of many of these viral proteases. In order to assess these similarities and differences, the structurally-similar proteins reported in Table 1 were used to generate both structure-based and sequence-based dendrograms (Figure 4). The structure-based dendrogram is based on structural similarity scores between the 3C-like proteases measured by DALI Z scores (summarized in Supplementary Figure S1), and the sequence-based dendrograms are constructed using Clustal Omega. Note that we avoid calling these “phylogenetic trees”, as it is not certain that all of the structural and sequence similarities between clades are a result of evolutionary divergence.

The structure-based dendrogram on 23 proteases (22 in Table 1, plus SARS-CoV2 3CL^pro^) clusters them into five structurally-similar clades (Figure 4, left), with 2 - 7 proteins per clade, plus three singleton clades. The pairwise Dali Z scores between members of each clade is > 20 (Supplementary Figure S1), indicating high structural similarity within each clade. Similar results were obtained using either domains I and II together, or when including also domain III in the DALI query. This structure-based dendrogram organizes clades consistently with the taxonomic classification of the corresponding viruses, with a clear bifurcation of the clades from the two Phyla (i.e. Phylum *Pisuviricota*, Orders *Picornavirales* and *Nidovirales* vs. Phylum *Kitrinoviricota*, Order *Amarillovirales*). All of the proteins from viruses within a common Order cluster into related clades, each clade including all the proteins from viruses within the same family. Specifically, the five multimember structural-similarity clades of Figure 4 correspond to 5 virus families: *Picornaviridae*, *Caliciviridae*, *Arteriviridae*, *Coronaviridae*, and *Flaviviridae*. One of the singleton clades, containing HCV NS3/4A, corresponds to the *Hepacivirius* subfamily, of the *Flaviviridae* Family. A second singleton, containing the Cavally Virus (CV) 3CL^pro^ belongs to Family *Mesoniviridae* within the order *Nidovirales*, and locates in the dendrogram with other proteins from *Nidovirales* viruses. The third singleton, containing the TEV 3CL protease, belongs to a distinct Class *Stelpaviricites* within the Phylum *Pisuviricota*. Hence, the structure-based dendrogram in Figure 4 largely recapitulates the taxonomic relationships of the viruses associated with these proteins; the top three clades correspond to families in the Order *Picornavirales* (excluding TEV which belongs to a different taxonomic class), the next three clades down belong to the Order *Nidovirales*, and the bottom two clades belong to a distinct Phylum, *Kitrinoviricota*, and Order *Amarillovirales* (Family *Flaviviridae*, Genus *Flavivirus* and *Hepacivirus*). Since the three-dimensional structure of a protein is an important phenotypic feature with functional implications for evolutionary selection, it is not surprising that there is a close correlation between the structure-based dendrogram and the corresponding viral taxonomy.

Viral proteases that are structurally closest to the SARS-CoV2 3CL^pro^ (PDB id 6Y2G chain A) all come from viruses in the Order *Nidovirales* (Families *Coronaviridae*, *Mesoniviridae*, and *Arteriviridae*). These proteases all have a third domain, domain III, in addition to the two domains forming the double β-barrel fold. The third domain of the 3C-Like protease of Cavally Virus (CV) is quite similar to the third domain of SARS-CoV2 3CL^pro^, while the third domains of the NSP4 proteinase from the Equine Arteritis Virus (EAV) (PDB id 1MBM) and the 3CL protease of Porcine Reproductive and Respiratory Syndrome Virus (PRRSV) (PDB id 5Y4L) are structurally different. The 3CL proteases from viruses of Order *Picornavirales* [e.g., human Rhinovirus (RHV), foot-and-mouth disease virus (FMDV), hepatitis A virus (HAV), and human Norovirus (NOV)] and Order *Amarillovirales* [e.g., dengue virus (DENV), West Nile virus (WNV), Zika virus (ZKV), and hepatitis C virus (HCV)] all have only domains I and II of the double beta-barrel fold, without the additional domain III.

Figure 4 (right) also shows a sequence-based dendrogram of these viral proteases. Generally, the sequence-based dendrogram is similar to the structure-based dendrogram, identifying the same five multiprotein clades. However, in this case the neat relationships between clades and taxonomic classification is absent. Relative to the structure-based dendrogram, the sequence-based dendrogram mixes clades between taxonomic classes. For example, the top three clades, with some sequence similarity to one another, belong to the taxonomic Families *Caliciviridae* (pink) of Order *Picornavirales*, *Flaviridae* (orange) of Order *Amarillovirales*, and *Arteriviridae* (yellow) of Order *Nidovirales*. While the *Picornaviridae* and *Caliciviridae* families of Order *Picornavirales* are recognized as individual clades, they are remote in the sequence-based dendrogram. Similarly, the *Coronaviridae* and *Arteriviridae* families are recognized as clades of Order *Nidovirales* but are also remote in the dendrogram. HCV NS3/4A protein forms an independent clade, and its structural and taxonomic relationship to other proteins from viruses in the Family *Flaviridae* is not evident in this sequence-based dendrogram. In addition, the CV 3CL protease, a virus in the taxonomic Order *Nidovirales*, is classified as a singleton, with no indication of its taxonomic and structural relationship to proteins from other *Nidovirales* viruses.

This disconnect between taxonomy and sequence-based dendrograms is attributable in part to the very low sequence similarity of proteases between families. Considering this low sequence similarity, one explanation for the structural similarity of substrate binding sites and superimposition of catalytic residues of these proteases from different taxonomic families is that they have converged in evolution on a common three-dimensional structure in order to achieve similar biochemical functions. For example, despite their common active site, substrate binding cleft, and sensitivity to several protease inhibitors, there is no phylogenetic evidence for common ancestors of HCV NS3/4A protease and SARS-CoV2 3CL^pro^, or of HCV and SARS-CoV viruses. Indeed, these two viruses belong to different taxonomic Phyla (Figure 3). The HCV NS3/4A protease is a serine-protease, with catalytic triad His57, Asp81, and Ser139, while the SARS-CoV2 3CL^pro^ is a cysteine protease, with catalytic dyad residues His41, Cys145 (Figure 1), consistent with the concept of convergent evolution to achieve a similar proteolytic function.

### Fold topology analysis

**FIGURE 5:**
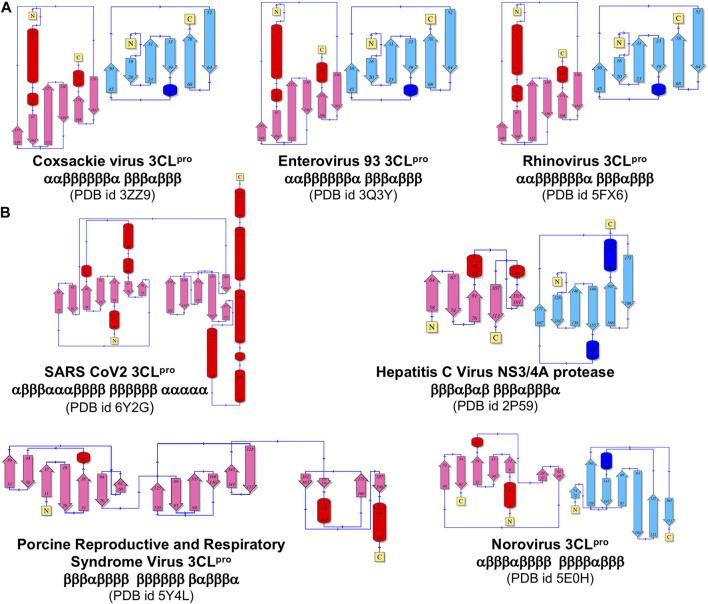
Comparison of topological representation of secondary structural elements of viral protease. **(A)** Topology of proteases of the viruses belonging to the same Picornaviridae Family. **(B)** Topology of proteases from different Families listed in Figure 3. ⍺-Helices are represented as cylinders and β-sheets are represented as arrows. These topological diagrams were obtained from PDBsum (http://www.ebi.ac.uk/pdbsum) (Laskowski et al., 2018), with the indicated PDB id for each protease.

Similarities in overall fold, locations of substrate binding sites, and common positioning of active-site residues can result from either homologous (divergent) evolution, or by convergence of different lineages to a common structure in order to achieve similar functions. In order to test the hypothesis that the observed disconnect between the structure- and sequence-based dendrograms (Figure 4) is due to convergent evolution of these proteins, we carried out a detailed fold topology analysis (Figure 5). Fold topology refers to the order of secondary structure elements within super-secondary structure or domains, and how these secondary structures are connected along the protein polypeptide chain (Thornton et al., 1999). Evolutionary divergence generally preserves, or changes in simple ways (including circular permutations or chain swapping) the fold topology, while proteins with very different topologies but similar functions can arise from different evolutionary lineages. Supporting the convergent evolution hypothesis for these 3C-like protease families, we observe that the several proteins within each of the five multimember structure-based clades have very similar fold topologies (Figure 5A, and Supplemental Figures S2–S6), while structures in different clades (corresponding to different taxonomic families) have quite different fold topologies (Figure 5B). Proteins from clades/families of the same taxonomic order are more similar to one another. One interesting exception is the striking similarity in the fold topologies of domain II of 3C-like proteases from virus Orders *Picornavirales* (e.g., Coxsackie virus 3CL^pro^) and *Amarillovirales* (e.g., Hepatitis C Virus NS3/4A protease) (Figure 5), which belong to different Phyla (Figure 4). Hence, for the 3C-like proteases, similarities in structural topology, like similarities in overall 3D structures, follow more closely the taxonomic classification of the corresponding viruses than sequence similarity metrics.

### Docking simulations with HCV NS3/4A protease inhibitors

The results outlined above suggest that structural similarity across viral 3C-like proteases may provide a basis for broad spectrum activities of 3C-like protease inhibitors. In previous studies, we assessed the use of *AutoDock* with flexible ligand conformation and fixed protein receptor conformation for inhibitor docking studies with SARS-CoV2 3CL^pro^. *The aim of these docking studies is not to necessarily predict an accurate binding pose, but rather to provide supporting data on the feasibility for proposed inhibitors to bind into the substrate binding and/or active site of SARS-CoV2 3CL* ^*pro*^ *.* Many of the molecules found to bind SARS-CoV2 3CL^pro^ with good *AutoDock* scores were subsequently observed to inhibit the enzyme activity *in vitro*, and in some cases to also inhibit viral replication in cell-based assays (Bafna et al., 2020; Ma et al., 2020; Bafna et al., 2021; Gammeltoft et al., 2021; Lo et al., 2021). For several cases where X-ray crystal structures of small molecule—3CL^pro^ complexes are available, we consistently observed *AutoDock* docking poses with an excellent match to the crystal structures among the best-scoring docked states (Bafna et al., 2021).

Based on the bioinformatics analysis outlined above, additional docking simulation calculations were carried out for several HCV NS3/4A protease inhibitor drugs using a similar protocol, with a larger docking grid size that used in our previous work (Bafna et al., 2020; Bafna et al., 2021) to accommodate larger peptide-like inhibitor molecules. These molecules, summarized in Table 2, have all been approved for at least Phase 1 clinical trials; some are FDA approved prescription drugs useful in treating hepatitis C virus infection. Results are also provided in Table 2 for the SARS-CoV2 inhibitor 13b. The *AutoDock* scores of the best scoring pose (i.e., lowest *AutoDock* binding energy) for each of these 12 HCV NS3/4A protease inhibitors are also summarized in Table 2. All of these 12 molecules, with *AutoDock* scores ranging from -10.36 to -13.79 kcal/mol, have more favorable binding scores than the α-ketoamide inhibitor 13b known to inhibit SARS-CoV2 3CL^pro^; *AutoDock* score 10.69 kcal/mol for best-scoring pose which is also the pose that best matches to the crystal structure of this complex (PDB id 6Y2G) (Zhang et al., 2020).

**TABLE 2:** Docking scores for HCV 3C/4A protease inhibitors with SARS-CoV2 3CL^pr^.^o^.

Inhibitor (Trade Name)	Identifier of protease inhibitor	Database id of protease inhibitor structure	*AutoDock* score (kcal/mol) Lowest “Energy”	Drug Status
**SARS-CoV2 3CL** ^**pro**^ **inhibitor**				
α-ketoamide inhibitor lowest “energy” pose pose most similar to X-ray structure	13b	6Y2G^a^	−10.69	Not Applicable
**HCV NSP3/4AProtease Inhibitor Drugs**				
Paritaprevir (Veruprevir/ABT-450; Abbot)	PAR	32700634^b^	−13.79	Prescription Drug
Narlaprevir (Arlansa; Merck/R-Pharm)	NAR	3LON^c^	−13.36 (−10.40) *	Prescription Drug
Boceprevir (Victrelis; Merck)	BOC	DB08873^d^	-13.17 (-11.44) *	Prescription Drug
Sovaprevir (ACH-1625; Achillion)	SOV	28529313^b^	−13.16	Investigational
Glecaprevir (Mavyret^e^/Maviret^e^; AbbVie/Enanta)	GLE	35,013,015^b^	−13.01	Prescription Drug
Simeprevir (Olysio; Medivir/Janssen)	SIM	3KEE^c^	−12.19	Prescription Drug
Telaprevir (Incivek/Incivo; Vertex/J&J)	TEL	3SV6^c^	−12.02	Prescription Drug
Danoprevir (Ganovo; Array/Pfizer, Roche/Ascletis)	DAN	3M5L^c^	−11.65	Investigational
Faldaprevir (Fadaprevir, Boehringer-Ingelheim)	FAL	3P8N^c^	−11.49	Investigational
Asunaprevir (Sunvepra; Bristol-Myers Squibb)	ASU	4WF8^c^	−11.46	Investigational
Grazoprevir (Zepatier; Merck)	GRZ	3SUD^c^	−10.77	Prescription Drug
Vaniprevir (MK-7009; Merck)	AN	3SU3^c^	−10.36	Investigational

a Atomic coordinates for the inhibitor taken from 6Y2G.

b Atomic coordinates for the inhibitor were taken from the ChemSpider database.

c Atomic coordinates for the inhibitor were taken from the PDB, coordinates of the corresponding complex of the inhibitor bound to HCV, 3C/4A protease.

d Atomic coordinates for the inhibitor were taken from the DrugBank database. * *AutoDock* score for pose most similar to the X-ray crystal structure.

e Mavyret (or Maviret) is a multidrug formulation including glecaprevir and pibrentasvir.

While *AutoDock* scores (Table 2) are useful for assessing the feasibility of complex formation, they are not sufficiently accurate to correctly rank the observed activities of these HCV drugs as inhibitors of SARS-CoV2 3CL^pro^. However, seven of these HCV proteases including narlaprevir (NAR), boceprevir (BOC), simeprevir (SIM), telaprevir (TEL), asunaprevir (ASU), grazoprevir (GRZ), and vaniprevir (VAN), do in fact inhibit SARS-CoV2 3CL^pro^ enzyme activity with IC_50_ of 2–50 μM, and also inhibit viral replication in Vero or human cells in similar concentration ranges (Bafna et al., 2021; Gammeltoft et al., 2021). Hence, the *AutoDock* scores for HCV drugs binding and inhibiting SARS-CoV2 3CL^pro^ have useful prognostic value in identifying lead molecules for testing and optimization. Surprisingly, three of these HCV drugs SIM, GRZ, and VAN, along with HCV protease inhibitor drug paritaprevir (PAR), also inhibit the SARS-CoV2 papain-like protease (PL^pro^), providing an alternative pathway for inhibition of SARS-CoV2 viral replication in cell culture (Bafna et al., 2021).

For all 12 drugs, the SARS-CoV2 3CL^pro^ bound-state pose with best *AutoDock* score fits well in the active site of the enzyme and recapitulates many of the key ligand-protein interactions observed in the complex with α-ketoamide inhibitor 13b (Supplementary Table S1). Some of these predicted drug—SARS-CoV2 3CL^pro^ complexes are shown in Supplementary Figures S7, S8. In this analysis, we paid particular attention to key details of the docking conformations, including interactions with the side chains of catalytic dyad residues His41 and Cys145, and hydrogen-bonded interactions with the backbone amides of Gly143, Ser144, and Cys145, which form the oxyanion hole of this cysteine protease (13). X-ray crystal structures are also available for boceprevir (BOC), narlprevir (NAR), and telaprevir (TEL) bound to SARS-CoV2 3CL^pro^ (Fu et al., 2020; Kneller et al., 2020; Oerlemans et al., 2020; Kneller et al., 2021a; Bai et al., 2021; Kneller et al., 2021b). Although the drug poses in these crystal structures are somewhat different than in the corresponding lowest-energy *AutoDock* poses, they include some of the same ligand—protein interactions (Supplementary Table S1). These modeling predictions further support the premise that inhibitors of one member of the viral PA superfamily (e.g., inhibitors of HCV protease) have the potential to function as lead molecules for development of inhibitors of other enzymes in this family (e.g., SARS-CoV2 3CL^pro^), even though these enzymes do not appear to be homologs evolved by evolutionary divergence.

**FIGURE 6:**
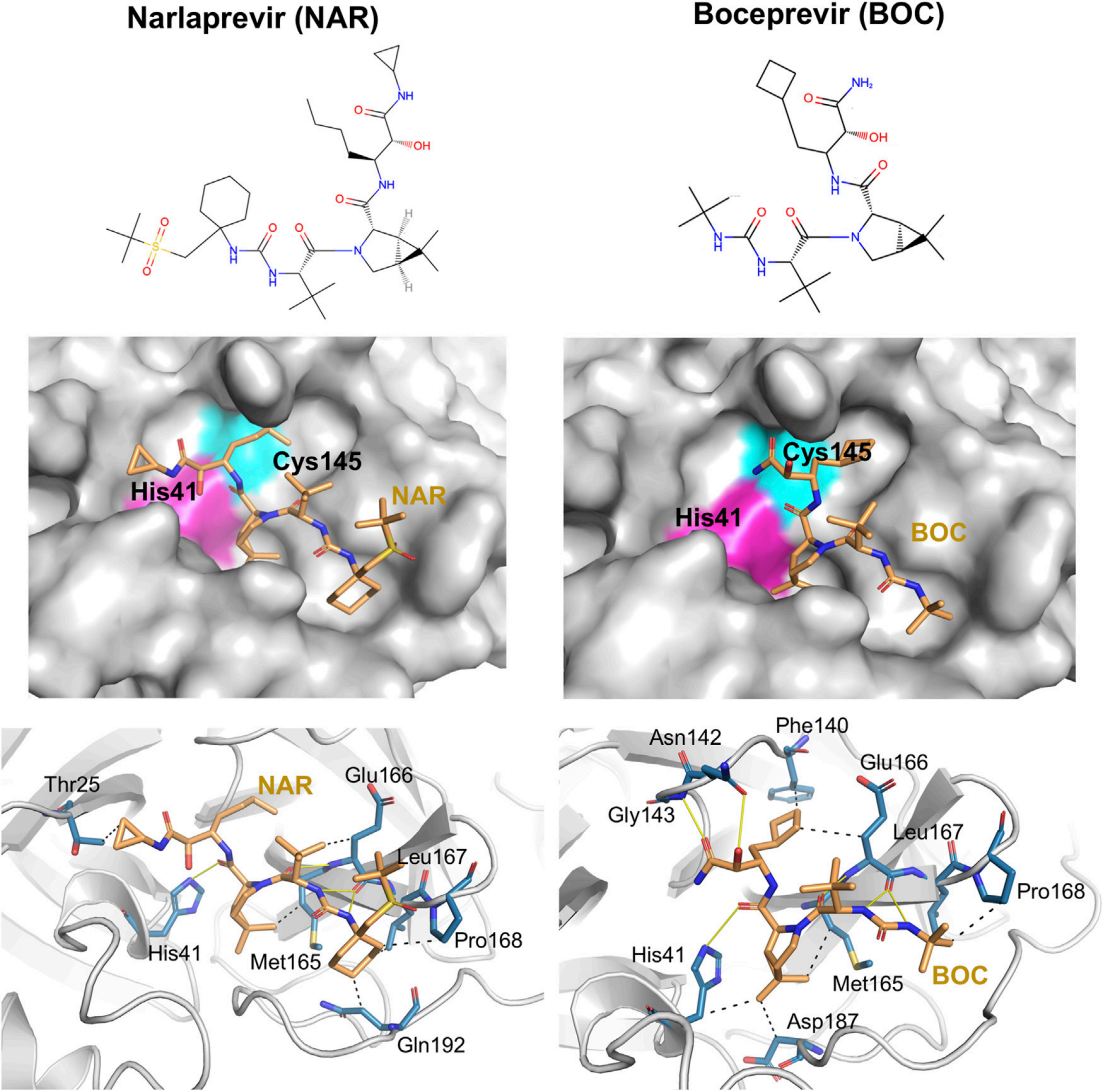
Docking of HCV protease NS3/4A inhibitor drugs to SARS-CoV2 3CL^pro^. Top panels - Molecular structures of two HCV protease inhibitor drugs. Middle panels—Lowest energy *AutoDock* pose of these HCV protease inhibitors (orange sticks) in the SARS CoV2 3CL^pro^ active site, Bottom panels—Details of atomistic interactions in the lowest energy *AutoDock* poses of these HCV protease inhibitors. Hydrogen bonds and hydrophobic interactions between the drug and the enzyme are shown with yellow solid lines and black dashed lines, respectively. Sidechains of catalytic residues His41 and Cys145 are labeled, along with other protein residues that form key interactions with these drugs.

**FIGURE 7:**
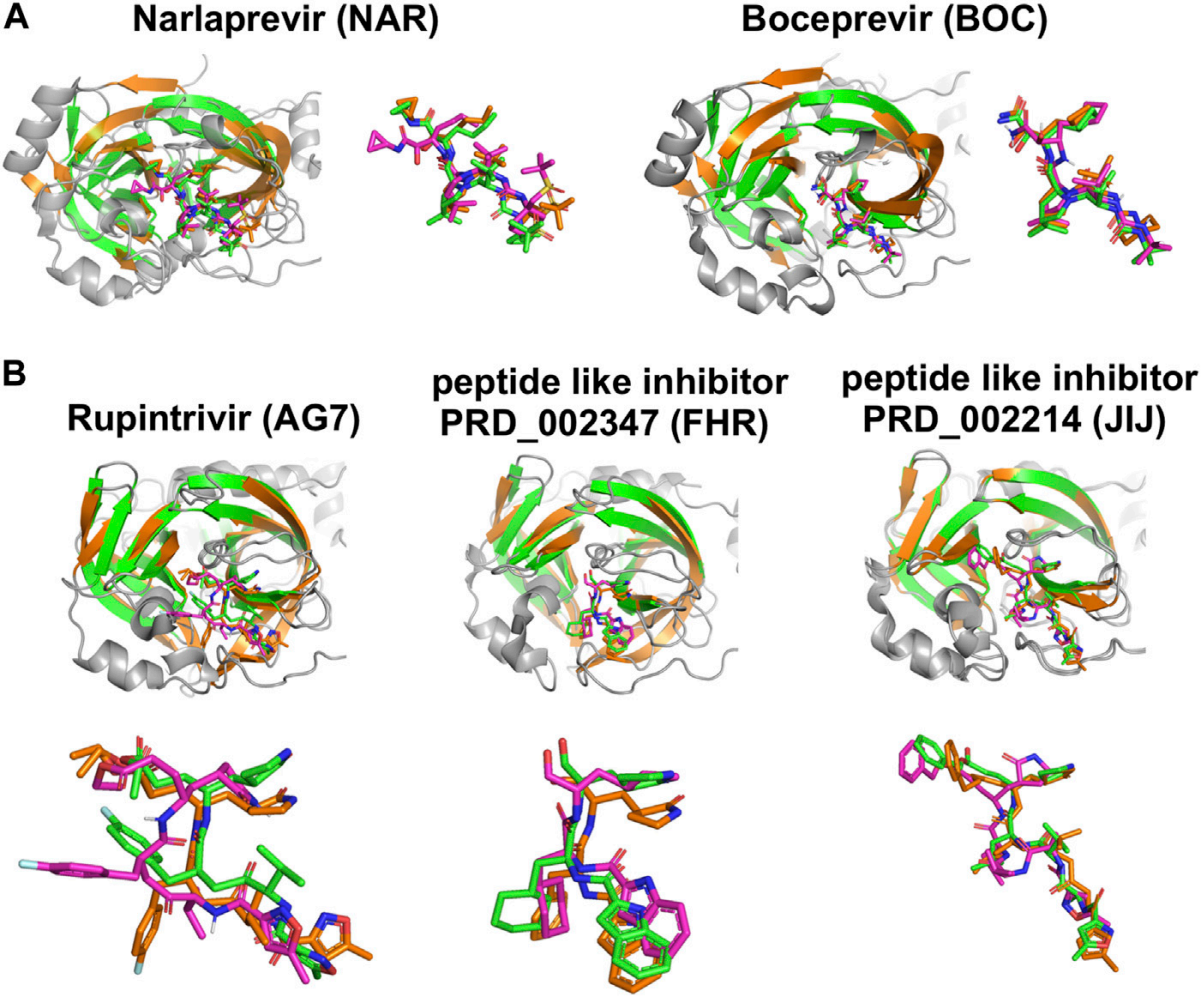
Comparisons of experimentally-determined structures and predicted docking poses of drugs and inhibitors bound to SARS-CoV2 3CL^pro^ and other viral proteases. **(A)** Comparison of the HCV NS3/4A protease inhibitors NAR and BOC binding pose in *AutoDock* (magenta) with the X-ray crystal structure in complex with SARS-CoV2 3CL^pro^ (green, NAR (PDB: 6XQT) and BOC (PDB: 6XQU)) and X-ray crystal structure in complex with HCV NS3/4A protease (orange, NAR (PDB: 3LON) and BOC (PDB: 3LOX)). **(B)** Comparison of inhibitor binding poses in *AutoDock* (magenta) with, X-ray crystal structures of complexes with SARS-CoV2 3CL^pro^ (green, AG7 (PDB: 7L8J), FHR (PDB: 6LZE), N3 (PDB: 7BQY)) and X-ray crystal structure in complex with other proteases (orange, HEV93-AG7 (PDB: 3RUO), HEV71-FHR (PDB: 7DCN), MHV-N3 (PDB: 6JIJ)). *AutoDock* poses most similar to crystal structure pose are shown here.

Details of intermolecular interactions for the *AutoDock* modes of NAR and BOC bound to SARS-CoV2 3CL^pro^ that are most similar to the corresponding X-ray crystal structures are illustrated in Figure 6. These binding poses exhibit extensive hydrogen-bonded and hydrophobic interactions within the substrate binding site and have relatively low *AutoDock* energies of -10.40 and -11.44 kcal/mol, for NAR and BOC complexes, respectively. These predicted poses are compared to the corresponding X-ray crystal structures of these same drugs bound to SARS-CoV2 3CL^pro^ and HCV NS3/4A proteases in Figure 7A. These binding modes of BOC and NAR in these two inhibitor—SARS-CoV2 3CL^pro^ complexes are also very similar to those observed in the crystal structures of the corresponding complexes with HCV NS3/4A protease (Bennett et al., 2010; Bai et al., 2021). The binding of TEL to SARS-CoV2 3CL^pro^ requires structural changes in the protease (Kneller et al., 2020), and this binding mode is not so well predicted by *AutoDock*.

### Novel 3CL^pro^ inhibitor predictions

SARS-CoV2 3CL^pro^ is more structurally similar to each of the 22 viral proteases listed in Table 1 than it is to the HCV NS3/4A protease (Table 1 and Figure 2). Considering the high success in identifying SARS-CoV2 3CL^pro^ inhibitors based on its structural similarity with HCV NS3/4A protease (Bafna et al., 2021), and having established the value of these *AutoDock* protocols in predicting potential small molecule—3CL^pro^ complex structures and providing useful hypotheses for lead development, we carried out the same docking protocol on 51 known inhibitors of the 22 viral proteins listed in Table 1. These results are summarized in Table 3. Interestingly, 19 of these molecules have *AutoDock* scores equal to or better than the scores for the several HCV drugs and the 13b inhibitor previously shown to inhibit both SARS-CoV2 3CL^pro^ enzymatic activity and viral replication in cell culture at low micromolar concentrations (Zhang et al., 2020; Bafna et al., 2021; Gammeltoft et al., 2021), including VAN (*AutoDock* score −10.36 kcal/mol).

**TABLE 3:** Docking scores for viral protease inhibitors with SARS-CoV2 3CL^pr^.^o^.

Inhibitor name	Database id of protease inhibitor structure	Identifier of protease inhibitor	*AutoDock* score (kcal/mol) Lowest “Energy”	Type of binding in crystal structure	Protease Target(s)
Nelfinavir	7DOZ (Bihani et al., 2021)	1UN	−13.16	non-covalent	Dengue virus NS2B/NS3 protease
Triazole-based macrocyclic inhibitor	5E0J (Weerawarna et al., 2016)	5LJ	−12.49	covalent^a^	Norovirus 3C-Like protease
Triazole-based macrocyclic inhibitor	6BID (Galasiti Kankanamalage et al., 2019)	DW4	−11.82	covalent	Norovirus 3C-Like protease
⍺,β -unsaturated ethyl ester inhibitor	3ZZA	G84	−11.57	covalent	Coxsackievirus B3 3C-Like protease
Compound 15	6KK5 (Braun et al., 2020)	DE6	−11.19	non-covalent	Zika virus NS2B/NS3 protease
Analog of Rupintrivir (γ-phenyl substitution)	5FX6 (Kawatkar et al., 2016)	6OY	−11.09	covalent	Rhinovirus 3C-Like protease
Compound 4	6KK3 (Braun et al., 2020)	DUU	−11.06	non-covalent	Zika virus NS2B/NS3 protease
AG7404	3Q3Y (Costenaro et al., 2011)	XNV	−10.98	covalent	Human Enteroviruses 3C-Like protease
Dipeptidyl inhibitor (Hexagonal form)	5T6G (Galasiti Kankanamalage et al., 2017)	N40	−10.96	covalent	Norovirus 3C-Like protease
Compound 8	6KPQ (Braun et al., 2020)	DT0	−10.91	non-covalent	Zika virus NS2B/NS3 protease
Bromocriptine	7JVR (Zhuang et al., 2021)	08Y	−10.77	non-covalent	Zika virus NS2B/NS3 protease
Rupintrivir (AG7088)	3RUO (Costenaro et al., 2011)	AG7	−10.60	covalent	Coxsackie virus A16 3C-Like protease Rhinovirus 3C-Like protease Human Enteroviruses 3C-Like protease
PRD_002347	6LZE (Dai et al., 2020)	FHR	−10.58	covalent	Human Enterovirus 71 3C-Like protease
Triazole-based macrocyclic inhibitor	6BIB (Galasiti Kankanamalage et al., 2019)	DW7	−10.55	covalent	Norovirus 3C-Like protease
⍺,β -unsaturated ethyl ester inhibitor	3ZZ9	G83	−10.48	covalent	Coxsackie virus B3 3C-Like protease
PRD_002214 (N3)	6JIJ (Cui et al., 2019)	JIJ	−10.48	covalent	Murine hepatitis virus strain A59 Main protease Porcine epidemic diarrhea virus (PEDV) 3C-Like protease Infectious bronchitis virus (IBV) Main protease
Compound 9	6KK4 (Braun et al., 2020)	DE0	−10.48	non-covalent	Zika virus NS2B/NS3 protease
Novobiocin	6B89 (May et al., 2017)	NOV	−10.39	non-covalent	Zika virus NS2B/NS3 protease
⍺,β -unsaturated ethyl ester inhibitor	3ZZB	G85	−10.38	covalent	Coxsackie virus B3 3C-Like protease
Compound16	6KK6 (Braun et al., 2020)	DV0	−10.35	non-covalent	Zika virus NS2B/NS3 protease
PRD 001171 (peptide inhibitor)	2M9Q	2M9	−10.32	covalent	Dengue virus NS2B/NS3 protease
Dipeptidyl inhibitor (Hexagonal form)	5T6F (Galasiti Kankanamalage et al., 2017)	N38	−10.23	covalent	Norovirus 3C-Like protease
⍺,β -unsaturated ethyl ester inhibitor	3ZZ8	G82	−10.19	covalent	Coxsackie virus B3 3C-Like protease
E22	5BPE (Zhai et al., 2015)	E22	−10.06	non-covalent	Human Enteroviruses 3C-Like protease
PRD_001054 (peptide inhibitor, syc59)	4INH (Muhaxhiri et al., 2013)	4IN	−10.06	covalent	Norwalk Virus Protease
Triazole-based macrocyclic inhibitor	5E0G (Guo et al., 2016)	5LG	−9.99	covalent	Norovirus 3C-Like protease
PRD_000568 (TG-0204998/0,204,998)	2ZU3 (Lee et al., 2009)	ZU3	−9.96	covalent	Coxsackie virus B3 3C-Like protease
PRD_002189 (oxadiazole-based, cell permeable macrocyclic (20-mer) inhibitor)	5DGJ (Damalanka et al., 2016)	V64	−9.96	covalent	Norovirus 3C-Like protease
Compound 9	5DP9 (Wu et al., 2016)	5EX	−9.94	covalent	Human Enteroviruses 3C-Like protease
⍺,β -unsaturated ethyl ester inhibitor	3ZZ6	G75	−9.91	covalent	Coxsackie virus B3 3C-Like protease
Allosteric inhibitor	6MO2 (Yao et al., 2019)	JVM	−9.89	non-covalent	Dengue virus NS2B/NS3 protease
Allosteric inhibitor	6MO0 (Yao et al., 2019)	JVJ	−9.79	non-covalent	Dengue virus NS2B/NS3 protease
PRD_000363 (Ace-LEALFQ-ethylpropionate inhibitor)	2B0F (Bjorndahl et al., 2007)	2B0	−9.74	covalent	Rhinovirus 3C-Like protease
Triazole-based macrocyclic inhibitor	6BIC (Galasiti Kankanamalage et al., 2019)	5LH	−9.67	covalent	Norovirus 3C-Like protease
Allosteric inhibitor	6MO1 (Yao et al., 2019)	I16	−9.63	non-covalent	Dengue virus NS2B/NS3 protease
PRD_001062 (peptide inhibitor, syc8)	4IMQ (Muhaxhiri et al., 2013)	4IM	−9.46	covalent	Norwalk Virus Protease
Compound 10	6Y3B (Braun et al., 2020)	O7N	−9.41	non-covalent	Zika virus NS2B/NS3 protease
Compound 2	6KK2 (Braun et al., 2020)	D9U	−9.31	non-covalent	Zika virus NS2B/NS3 protease
NK-1.8K	5GSO (Wang et al., 2017b)	5GI	−9.18	covalent	Coxsackie virus B3 3C-Like protease
Temoporfin	DB11630	TEM	−9.08	non-covalent	Zika virus NS2B/NS3 protease
macrocyclic inhibitor	6FFS (Namoto et al., 2018)	D8E	−9.06	covalent	Rhinovirus 3C-Like protease
⍺,β -unsaturated ethyl ester inhibitor	3ZZ7	G81	−8.81	covalent	Coxsackie virus B3 3C-Like protease
X77	6W81	X77	−8.79	non-covalent	Porcine epidemic diarrhea virus (PEDV) 3C-Like protease
dipeptidyl inhibitor (covalent)	4XBD (Galasiti Kankanamalage et al., 2015)	M40	−8.65	covalent	Norwalk Virus Protease
Carnosine	Pubchem 439,224	CAR	−8.11	non-covalent	Dengue virus NS2B/NS3 protease
MB21	88,296,444 Chemspider	MB2	−8.03	non-covalent	Dengue virus NS2B/NS3 protease
N-(iodoacetyl)-L-valyl-l-phenylalaninamide	1QA7 (Bergmann et al., 1999)	IVF	−7.66	covalent	Hepatitis A virus 3C proteinase
⍺,β-unsaturated ethyl ester inhibitor	3ZZ5	G74	−7.57	covalent	Coxsackie virus B3 3C-Like protease
2-phenylquinolin-4-ol (Non-covalent)	2XYA (Baxter et al., 2011)	7L4	−6.23	non-covalent	Rhinovirus 3C-Like protease
n-[(benzyloxy)carbonyl]-l-alanine (peptide-based ketone inhibitor)	2HAL (Yin et al., 2006)	BBL	−5.51	non-covalent	Hepatitis A virus 3C proteinase
NSC157058	Pubchem 423,738	NSC	−5.11	non-covalent	Zika virus NS2B/NS3 protease

a Molecules with the potential to form covalent complexes may bind significantly more favorably than indicated by relative *AutoDock* scores.

Chemical structures of the 20 top scoring inhibitors are shown in Supplementary Figure S9. Included in Table 3 are several molecules previously reported to inhibit SARS-CoV2 3CL^pro^ enzyme activity; viz compound PRD_002347 (FHR; *AutoDock* Score -10.58 kcal/mol), first reported as an inhibitor of human enterovirus 71 (HEV) 3C-like protease (Dai et al., 2022), compound PRD_002214 (N3 or JIJ; *AutoDock* Score—10.48 kcal/mol), reported as an inhibitor of murine hepatitis virus strain A59 (MHV) main protease (Cui et al., 2019), porcine epidemic diarrhea virus (PEDV) 3C-like protease (Wang F. et al., 2017), and avian infectious bronchitis virus (IBV) main protease (Wang F. et al., 2017), and compound X77 (*AutoDock* Score −8.79 kcal/mol) reported as an inhibitor of porcine epidemic diarrhea virus (PEDV) 3C-like protease (Mesecar, 2021). Rupintrivir (compound AG7088 or AG7, *AutoDock* Score −10.60 kcal/mol), an inhibitor of Coxsackie virus (CAV) A16 3C-like protease (Lu et al., 2011), human rhinovirus (RHV) 3C-like protease (Matthews et al., 1999), and human enteroviruses (HEV) 3C-like protease (Costenaro et al., 2011; Hung et al., 2011) is also reported to weakly inhibit SARS-CoV2 3CL^pro^ (Liu et al., 2021), although another study found that rupintrivir is not active against SARS-CoV2 3CL^pro^ (Ma et al., 2022). Nelfinavir, the best scoring molecule in Table 3, an inhibitor of the human dengue virus (DENV) NS2B/NS3 protease (Bhakat et al., 2015) is also a weak inhibitor of SARS-CoV2 3CL^pro^ (Ohashi et al., 2021). Comparisons of crystal structures determined for three of these inhibitors bound to SARS-CoV2 3CL^pro^ and one other viral 3C-like protease, together with low-energy *AutoDock* docking poses, are shown in Figure 7B. Taken together, the results support the view that inhibitors of viral proteases that are structurally-similar to SARS-CoV2 3CL^pro^ are valuable candidates for exploration as potential lead molecules for SARS-CoV2 3CL^pro^ inhibitor drug discovery programs.

## Discussion

The global health, economic, and social impact of the COVID-19 pandemic is enormous. Moreover, future pandemics, by coronaviruses or other pathogenic viruses, are inevitable. For example, disruptions to ecological niches due to global warming create the opportunity for emergent viruses to access new host ranges, increasing the prevalence of viral outbreaks. Although public health policies can slow the spread of a virus, effective control of viral diseases requires both vaccines and antivirals. In particular, antivirals are crucial for the present COVID-19 pandemic. Despite the initial success of the antiviral drug Paxlovid™, a combination of the 3CL^pro^ inhibitor nirmatrelvir and the P450 enzyme inhibitor ritonavir which improves the pharmacokinetics of nirmatrelvir, antiviral resistance is anticipated. Hence, the identification and development of additional orally-bioavailable inhibitors of SARS-CoV2 3CL^pro^ is critical. More generally, it is important to proactively develop an arsenal of antiviral drugs which can be used either individually or in combinations to suppress virus infection and avoid viral resistance.

**FIGURE 8:**
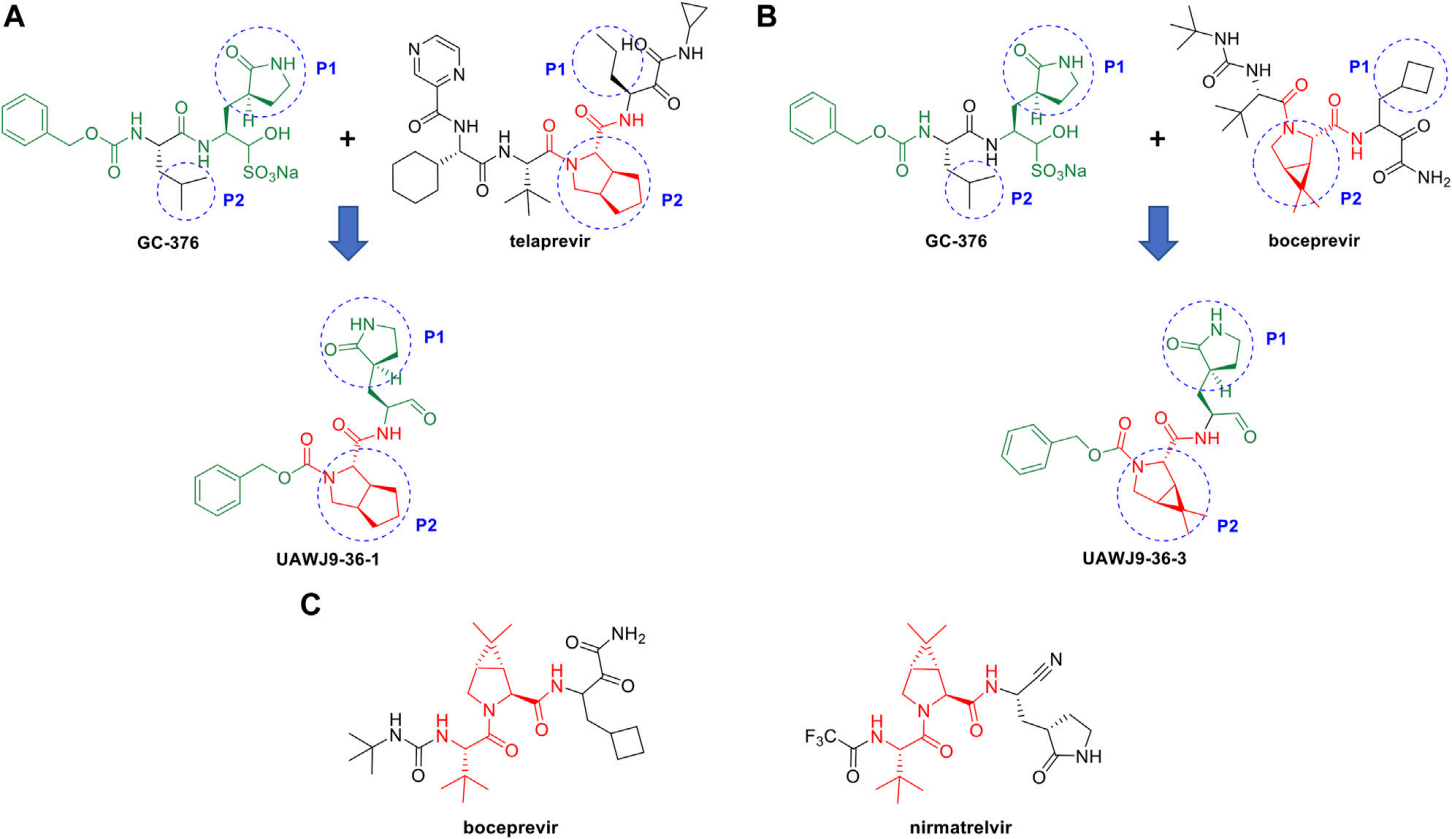
Rational design of novel SARS-CoV2 3CL^pro^ inhibitors using HCV NS3/4A protease inhibitor scaffolds. Structure-based drug design of novel SARS-CoV-2 3CL^pro^ inhibitors derived from GC-376 and the HCV protease NS3/4A inhibitors TEL **(A)** and BOC **(B)**. The structure of Pfizer’s SARS-CoV2 3CL^pro^ inhibitor nirmatrelvir has a core scaffold similar to BOC **(C)**.

In this context, repurposing of existing antiviral drugs previously developed for other viral 3C-like proteases, like those listed in Table 3, is vitally important and can quickly add to the armamentarium of SARS-CoV2 3CL^pro^ inhibitors. These newly identified compounds can also serve as viable leads with which to execute hit-to-lead and lead optimization drug discovery efforts towards novel SARS-CoV2 3CL^pro^ inhibitor chemotypes. For example, Wang and coworkers reported the discovery of novel and potent SARS-CoV2 3CL^pro^ inhibitors derived from the reported SARS-CoV2 3CL^pro^ inhibitor GC-376 and the HCV NS3/4A protease inhibitors TEL and BOC (Figures 8A,B) (Xia et al., 2021). Guided by X-ray co-crystal structures, the team generated novel hybrid chemotypes that exploited the overlay of key structural motifs. Notably, the superimposed X-ray co-crystal structures showed that the GC-376 leucine, TEL octahydrocyclopenta[c]pyrrole, and BOC 6,6-dimethyl-3-azabicyclo[3.1.0]hexane structures all occupy the hydrophobic S2 pocket, and it was anticipated that swapping the GC-376 leucine residue with the more lipophilic bicyclic core structures of TEL and BOC could potentially improve potency by engaging in additional hydrophobic interactions. These structural changes, along with incorporation of the GC-376 benzyl carbamate and other rational design modifications based on the overlaid structures, lead to the identification of two novel and promising SARS-CoV2 3CL^pro^ inhibitors (UAWJ9-36-1 and UAWJ9-36-3) that exhibit properties suitable for further development. Kneller et al., have also recently reported covalent hybrid inhibitors of 3CL^pro^ created by splicing components of hepatitis C protease inhibitors BOC and NAR, and known SARS-CoV1 protease inhibitors, which inhibit virus replication in cell culture (Kneller et al., 2022). Pfizer’s FDA-approved SARS-CoV2 3CL^pro^ inhibitor nirmatrelvir also contains the 6,6-dimethyl-3-azabicyclo[3.1.0]hexane and 2-amino-3,3-dimethylbutanamide structural elements of BOC (Figure 8C) (Zhao et al., 2021). These reports clearly demonstrate the significant value that identifying leads from existing protease inhibitors of other viruses can have for future SARS-CoV2 3CL^pro^ inhibitor drug discovery efforts.

The 3C-like proteases of *Orthornavirae* viruses, the kingdom of viruses with RNA genomes encoding an RNA-dependent RNA polymerase (RdRp), are essential to the virus life cycle, and are important targets for antiviral drug development. Koonin et al. first predicted that coronaviruses contain a protein (later identified as the main protease, 3CL^pro^) similar to the 3C proteases of picornaviruses (37), and Anand et al. (17) subsequently described the striking structural similarity between the active sites of transmissible gastroenteritis coronavirus (TGEV) 3CL^pro^ and the 3CL protease of pircornavirus hepatitis A virus (HAV). Our initial structural bioinformatics analysis (Bafna et al., 2020; Bafna et al., 2021) demonstrated strong structural similarity between the active sites SARS-CoV2 3CL^pro^ and HCV NS3/4A, which was surprising as these two enzymes are in very distantly related viruses (coronaviruses and flaviviruses, respectively) from different Phyla (see Figure 3). Most importantly, we and others have observed that inhibitors of HCV NS3/4A are also inhibitors of SARS-CoV2 3CL^pro^, and of SARS-CoV2 replication in cell culture (Bafna et al., 2020; Bafna et al., 2021; Gammeltoft et al., 2021; Lo et al., 2021).

Fold topology, overall fold, locations of substrate binding sites, and common positioning of active-site residues can result from homologous (divergent) evolutionary relationships between proteins. For example, 3C-like Cys proteases of picornoviruses have been proposed to be homologous to Ser proteases of the trypsin protease superfamily (Bazan and Fletterick, 1988). Both Koonin and co-workers (Gorbalenya et al., 1989) and James and co-workers (Allaire et al., 1994) have also proposed a divergent evolutionary relationship between the 3CL cysteine proteases of picornaviruses and chymotrypsin-like serine proteases. Coronaviruses and picornaviruses are in the same Class, but in different Orders. However, convergent evolution can also occur, and apparent structural convergence of protease active sites is a classic structural bioinformatics observation (Robertus et al., 1972; Kester and Matthews, 1977). The wide range of structural topologies observed across the positive-strand RNA proteases of the PA superfamily (Figure 5; Supplementary Figures S2—S4) support the idea that their analogous three-dimensional structures arose by evolutionary convergence on a common biochemistry rather than divergence from a common ancestor. In particular, the structural similarity in and around the active sites of the evolutionarily-distant HCV NS3/4A and SARS-CoV2 3CL^pro^ proteases is striking, and appears to be the result of convergent evolution from different fold topologies to create a similar binding pocket. Interesting in this regard, recent marine metagenomic sequencing and phylogenetic studies suggest that ancient ancestors of the positive-strand RNA viruses, including highly mobile RNA retroelements that can readily move to new locations in the genome, predate even the Last Universal Cellular Ancestor (LUCA) (Zayed et al., 2022). Such a pre-cellular RNA ecology could potentially provide a source of structurally-variable ancient progenitors of the various clades of modern 3C-like proteases of positive-strand RNA viruses.

Molecular docking is a widely used tool for modern structure-based drug discovery. It is used not only to explore the binding conformations of lead molecules in the active site of drug targets, but also to estimate the strength of interaction between the ligand and target. The *AutoDock* program used in our study offers a variety of search algorithms to recursively evaluate ligand conformations and uses a force-field-based scoring function to rank the binding poses. The accuracy of the program has been tested with a diverse set of protein–ligand complexes of biological and medicinal interest (Forli et al., 2016). The predicted *AutoDock* binding energies may not be highly accurate, and even relative affinities within a series of ligands cannot generally be reliably determined. While the best-scored *AutoDock* complex does not always match the experimentally determined structure, the experimentally determined structure is generally among the best scoring poses (Kolb et al., 2009; Kolb and Irwin, 2009). Accordingly, the best-ranked predictions, illustrated for example in Supplementary Figures S7 and S8, should capture key features of the ligand—protein interaction, but they might not be the dominant pose observed in future experimental studies.

Some known inhibitors of 3CL^pro^ form covalent bonds upon complex formation, which are not accounted for in these *AutoDock* models. For several cases where the three-dimensional structures of these covalent complexes are known, including complexes with compound 13b (Zhang et al., 2020), boceprevir (Kneller et al., 2020), and narleprevir (Kneller et al., 2020), covalent bond formation is in fact stabilizing one of the low energy *AutoDock* poses. As shown in Figures 6, 7, *AutoDock* calculations predict the crystal structure poses of these inhibitors where the respective alpha-ketoamide warhead is positioned to form a co-valent bond with the active site Cys thiol. Failure to account for covalent bond formation would not contradict the conclusion, based on good non-covalent docking scores, that a molecule is a potential inhibitor. However, appropriate consideration of covalent stabilization could rule in candidates with poorer non-covalent *AutoDock* scores. In order to address this, we identify also in Table 3 the proposed 3CL^pro^ inhibitors which potentially form covalent complexes with the active-site Cys residue. If such covalent bond formation occurs, these inhibitors could have enhanced binding affinity than indicated by simple *AutoDock* scores.

Another important limitation of the *AutoDock* protocol used here is the inability to model the conformational flexibility of the protein target. This problem is typically approached through the generation of multiple conformations of the protein by molecular dynamics before docking, or by allowing the ligand active site residues to be flexible during the docking runs, which are both important future direction for this work.

In conclusion, our studies describe interesting structural similarity between the 3C-like proteases of Kingdom *Orthornavirae* that comprises positive-stranded RNA viruses from multiple Classes and Phyla. The fact that the same molecules can inhibit SARS-CoV2 3CL^pro^ and HCV NS3/4A proteases, spanning the structural similarity scores and taxonomic distribution of proteases from a wide range of viruses in the Kingdom *Orthornavirae*, strongly supports the potential for considering inhibitors of this wide range of 3C-like proteases as lead molecules for developing novel broad spectrum viral protease inhibitor drugs.

## Data Availability

The authors acknowledge that the data presented in this study must be deposited and made publicly available in an acceptable repository, prior to publication. Frontiers cannot accept a manuscript that does not adhere to our open data policies.
