# Supplementary material for: Structural similarities between SARS-CoV2 3CLpro and other viral proteases suggest potential lead molecules for developing broad spectrum antivirals
Source: Front Chem. 2022 Oct 6;10:948553. doi: 10.3389/fchem.2022.948553 (PMC9638714; doi:10.3389/fchem.2022.948553)

Supplementary Materials

Supplementary Table 1. Key Interactions of HCV drugs within the SARS-CoV2 3CL^{pro} active site.

Inhibitor (Trade Name ; Manufacturer)	Identifier of Protease Inhibitor	Atoms Forming Key Hydrogen Bonds	Residues Forming Key Hydrophobic Interactions
SARS-CoV2 3CL Protease Inhibitor Drugs			
α-ketoamide inhibitor	13b	Gly143(N), Ser144(N,O^{γ}) , Cys145(N), His163(N^{ε}), His164(O), Glu166(N)	Met165, Glu166, Asp187, Gln189
HCV NS3/4A Protease Inhibitor Drugs			
Paritaprevir (ABT-450; Abbot)	PAR	His41(N^{ε}),Glu166 (N), Gln192(N)	His164, Met165, Glu166, Pro168, Asp187, Gln189
Narlaprevir (Arlansa; Merck / R-Pharm)	NAR	His41(N^{ε}), Asn142(O^{δ}), Gly143(N), Glu166 (N,O)	Phe140, Met165, Glu166, Leu167, Gln192
	NAR*	His41(N^{ε}),Glu166 (N,O)	Thr25, Met165, Glu166 Leu167, Pro168, Gln192
Boceprevir (Victrelis; Merck)	BOC	Gly143(N), Ser144(N,O^{γ}) , Cys145(N), His163(N^{ε}), His164(O), Glu166(O,N)	Met165, Glu166, Leu167, Asp187, Gln192
	BOC*	His41(N^{ε}), Asn142(O^{δ}), Gly143(N), Glu166(O,N)	His41, Phe140, Met165, Glu166, Leu167, Pro168, Asp187
Sovaprevir (ACH-1625; Achillion)	SOV	His41(N^{ε}), Gly143(N), Glu166(N), Gln189(N^{ε})	Thr25, Leu27, Leu167, Gln192
Simeprevir (Olysio; Medivir / Janssen)	SIM	Thr25 (O^{γ}), His41(O), Cys44(N), Glu166 (N)	Phe140,, Met165, Glu166, Gln189
Telaprevir (Incivek / Incivo; Vertex / J&J)	TEL	Glu166(O,N), Thr190(O), Gln192(N,N^{ε})	Met165, Glu166
Grazoprevir (Zepatier; Merck)	GRZ	Glu166(O), Gln192(N^{ε})	Leu141, Met165, Leu167, Gln189, Gln192
Vaniprevir (MK-7009; Merck)	VAN	Thr26 (N), Asn142(O^{δ}), Gly143(N), Ser144(N)	Phe140, Asn142,

* Docking pose closest to the crystal structure conformation

Figure S1: All-against-all structure similarity matrix for 22 selected viral proteases and SARS-CoV2 3CL^{pro}. The viral Families identified in structure-based dendrogram of Figure 4 are labeled. Each protease is labeled by their respective PDB id. Produced by DALI server (Holm 2020).

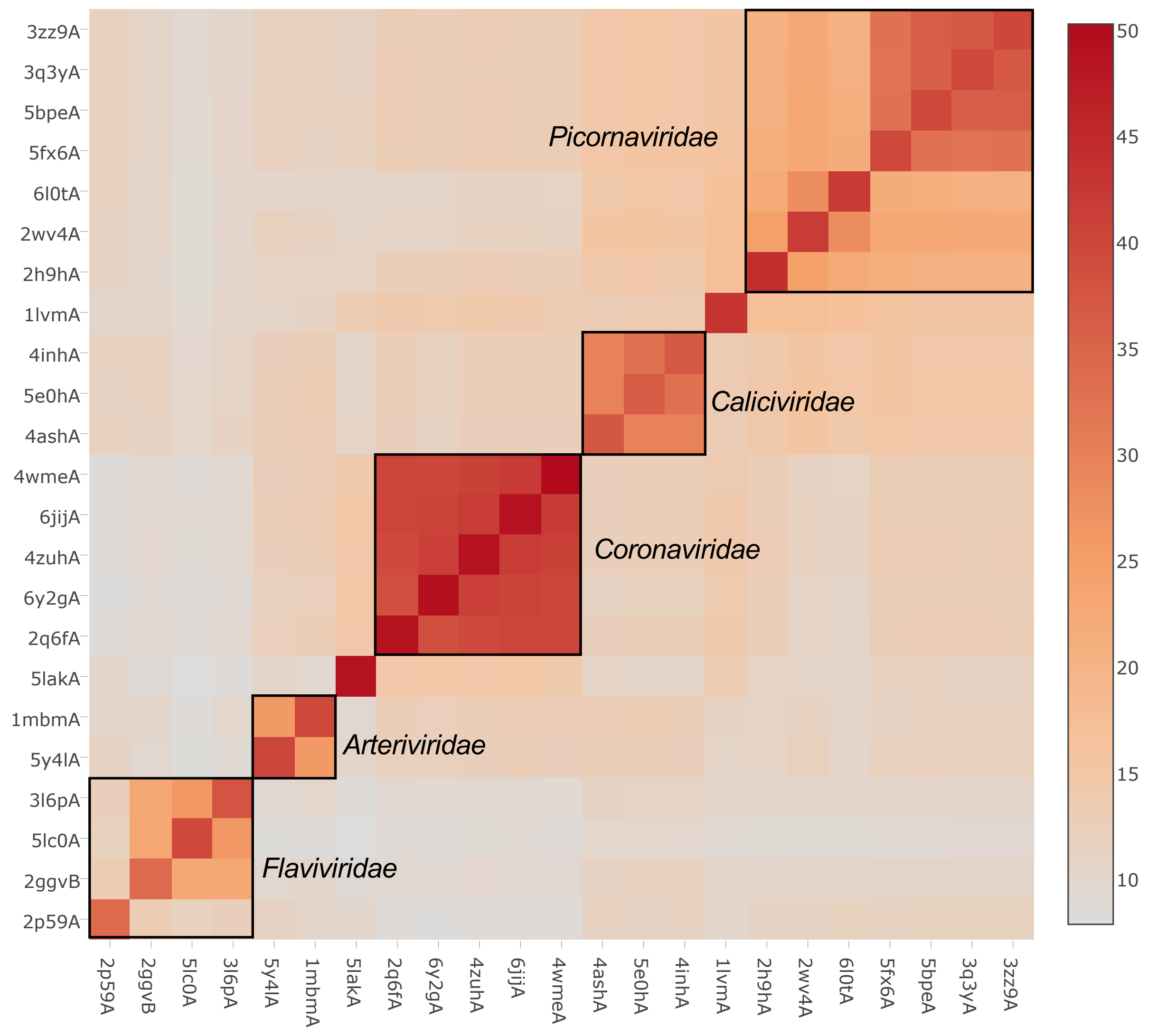

Figure S2: Comparison of topological representation of secondary structural elements of viral proteases of *Picornaviridae* Family. α-helices are represented as cylinders and β- sheets are represented as arrows. These topological diagrams were obtained from *PDBsum* (Laskowski et al, 2018) with the indicated PDB id for each protease.

Coxsackie virus 3CL^{pro}
ααββββββα βββαβββ
PDB id 3ZZ9

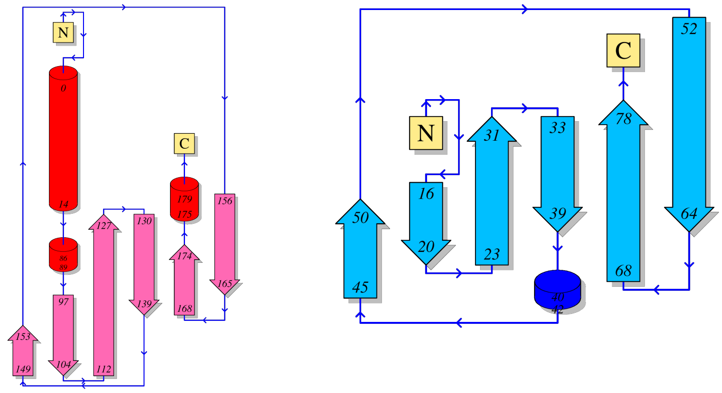

Enterovirus 93 3CL^{pro}
ααββββββα βββαβββ
PDB id 3Q3Y

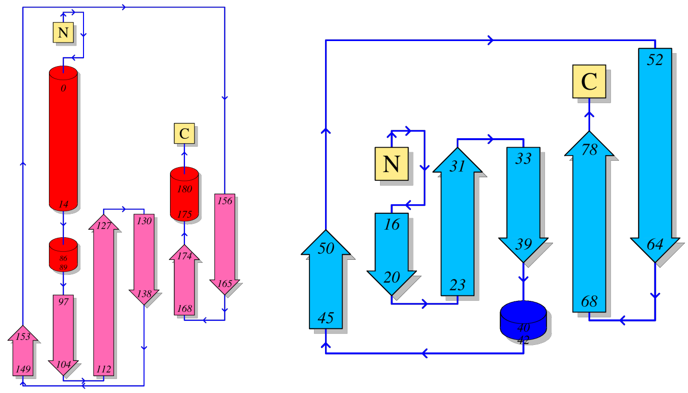

Enterovirus 71 3CL^{pro}
ααββββββα βββαβββ
PDB id 5BPE

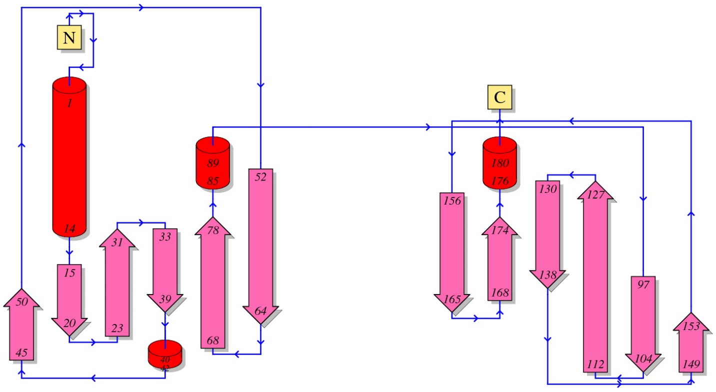

Rhinovirus 3CL^{pro}
ααββββββα βββαβββ
PDB id 5FX6

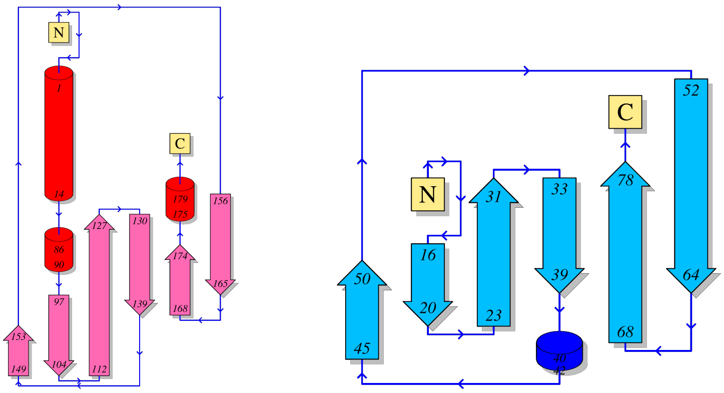

Fig S2 (con't)

Senecavirus A 3C pro
αβββαββββα βββαβββα
PDB id 6L0T

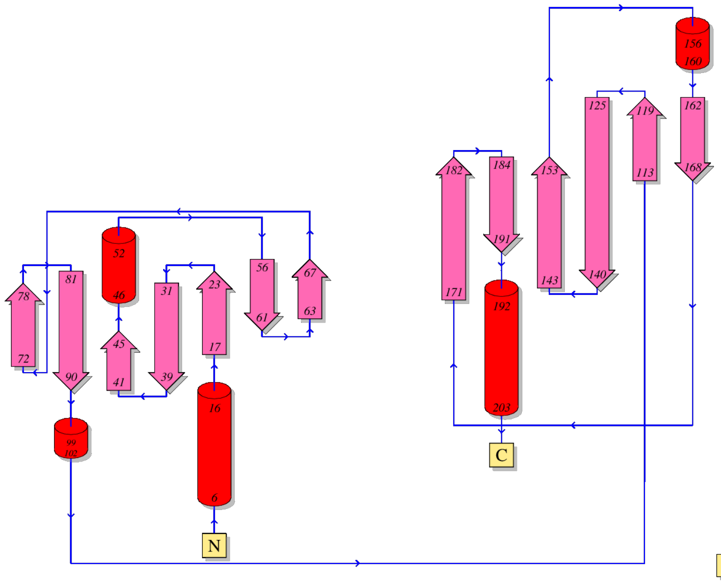

Foot and Mouth Disease 3C pro
αβββαββββββ αββββββα
PDB id 2WV4

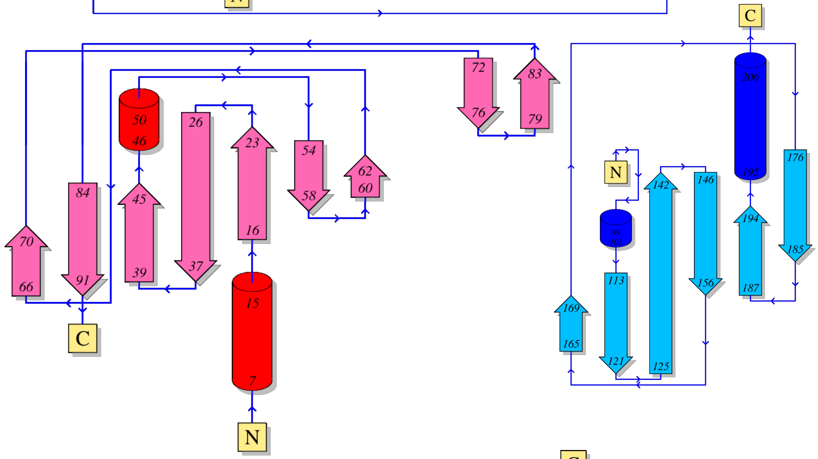

Hepatitis A Virus 3C pro
αβββααββαββα αβββββαββα
PDB id 2H9H

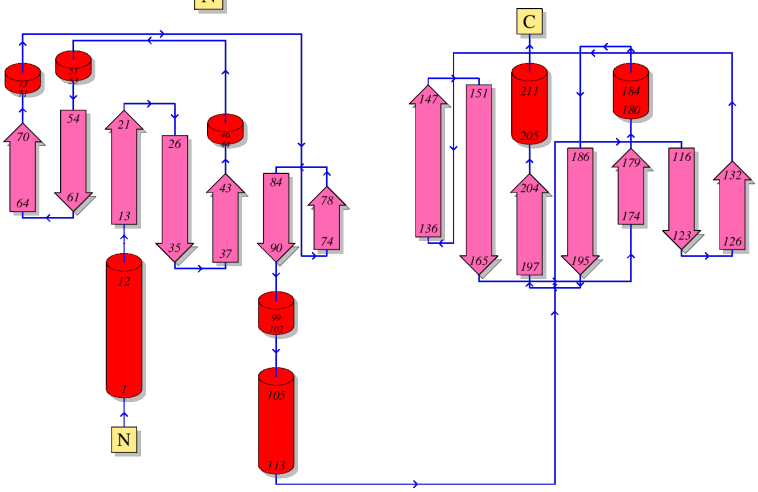

Tobacco Etch Virus 3CL pro
ααβββαββαββ ββββββββααβββ
PDB id 1LVM

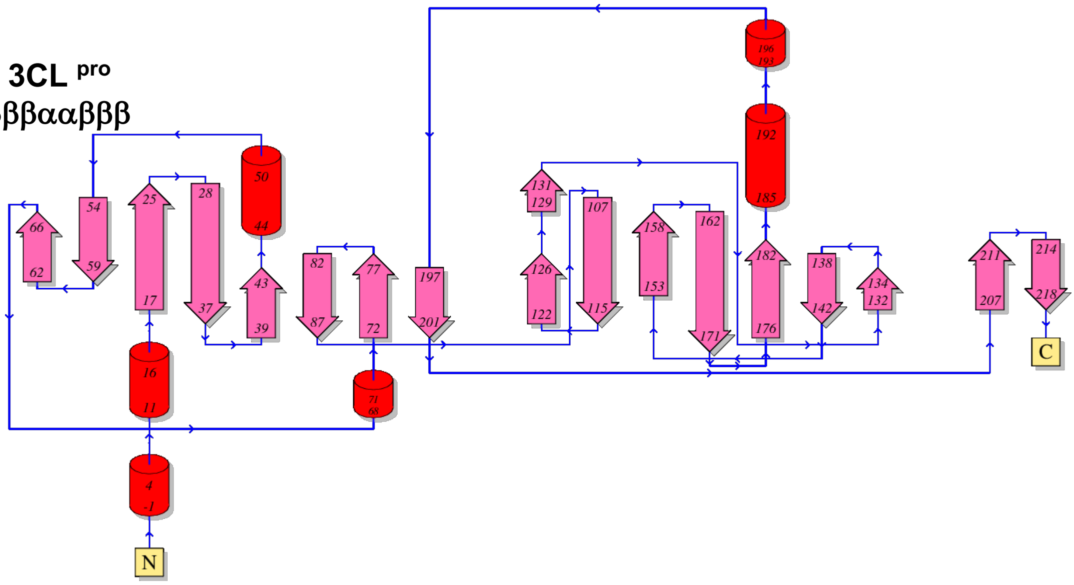

Figure S3: Comparison of topological representation of secondary structural elements of viral proteases of *Caliciviridae* Family. α-helices are represented as cylinders and β- sheets are represented as arrows. These topological diagrams were obtained from *PDBsum* (Laskowski et al, 2018) with the indicated PDB id for each protease.

Norwalk virus protease
αβββαββββ ββββαβββ
PDB id 4INH

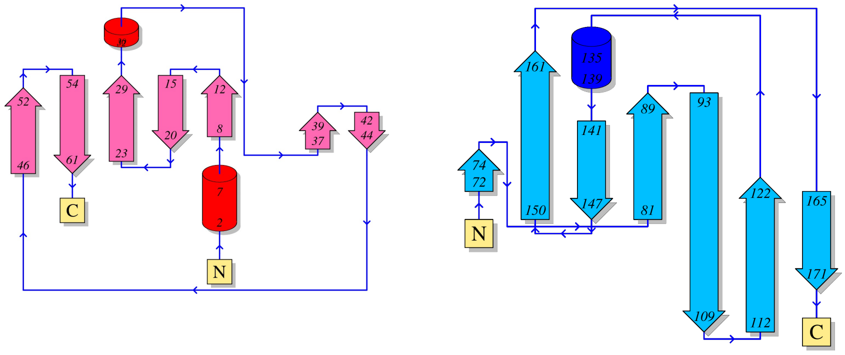

Norovirus 3CL [pro]
αβββαββββ ββββαβββ
PDB id 5E0H

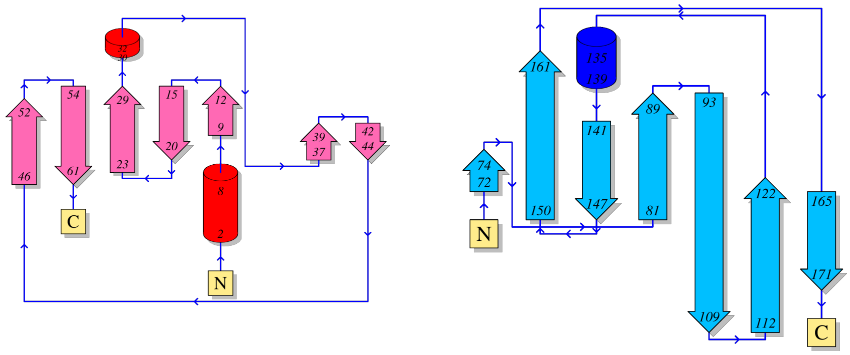

Murine Norovirus 1 NS6 protease
αβββαββ βββββββ
PDB id 4ASH

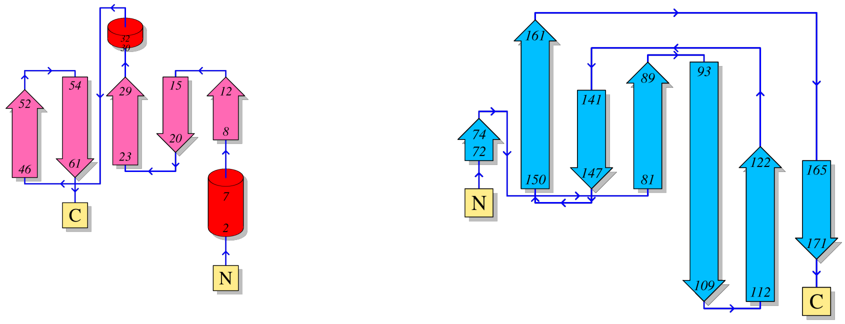

Figure S4: Comparison of topological representation of secondary structural elements of viral proteases of *Arteriviridae* Family. α-helices are represented as cylinders and β- sheets are represented as arrows. These topological diagrams were obtained from *PDBsum* (Laskowski et al, 2018) with the indicated PDB id for each protease.

Equine Arteritis Virus NSP4 proteinase

ββββαββββ βββαββαββ βαβββα

PDB id 1MBM

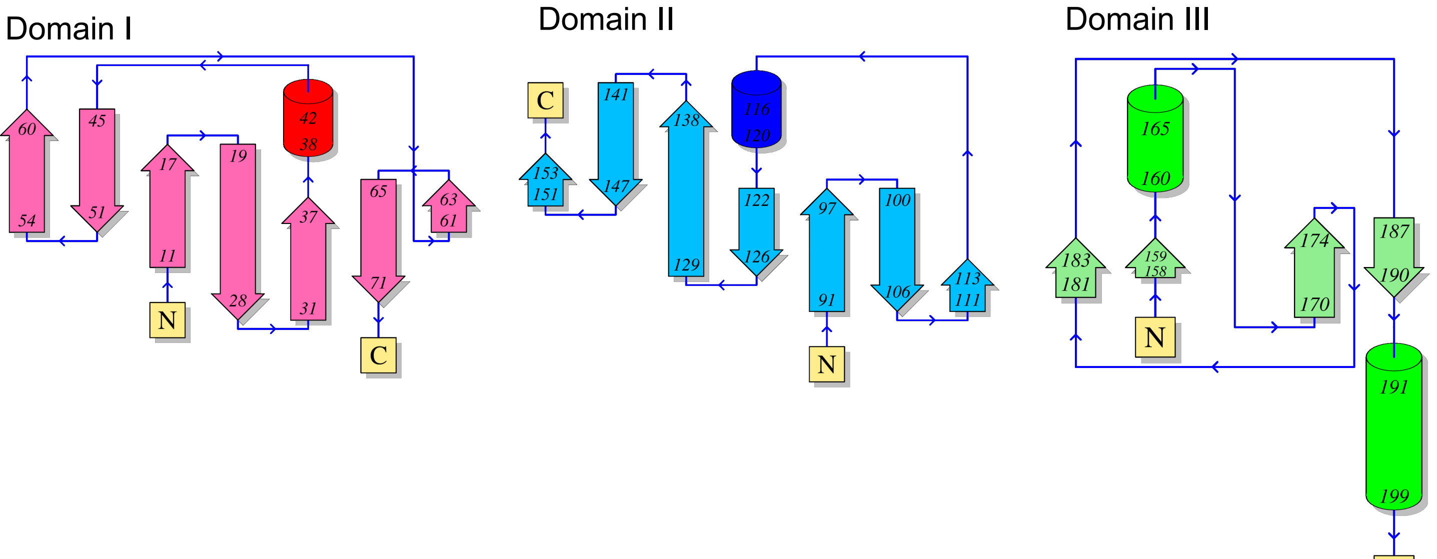

Porcine Reproductive and Respiratory Syndrome Virus 3CL pro

Βββαββββ ββββββ βαβββα

PDB id 5Y4L

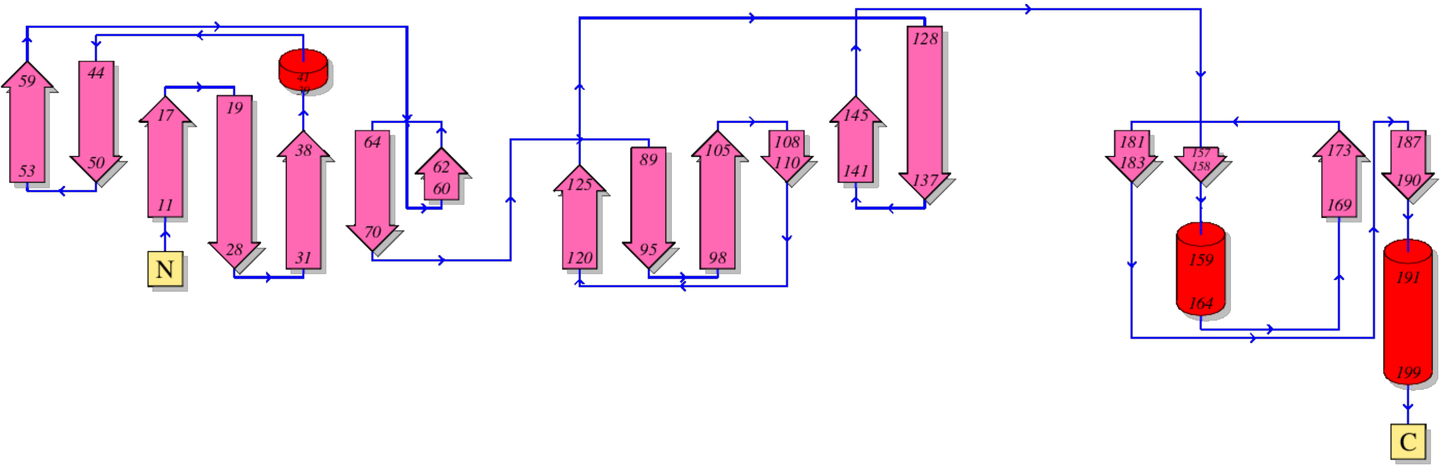

Figure S5: Comparison of topological representation of secondary structural elements of viral proteases of *Coronaviridae* Family. α-helices are represented as cylinders and β- sheets are represented as arrows. These topological diagrams were obtained from *PDBsum* (Laskowski et al, 2018) with the indicated PDB id for each protease.

Murine Hepatitis Virus Strain A 59 3CL pro
αβββαααββββ ββββββ ααααα
PDB id 6JIJ

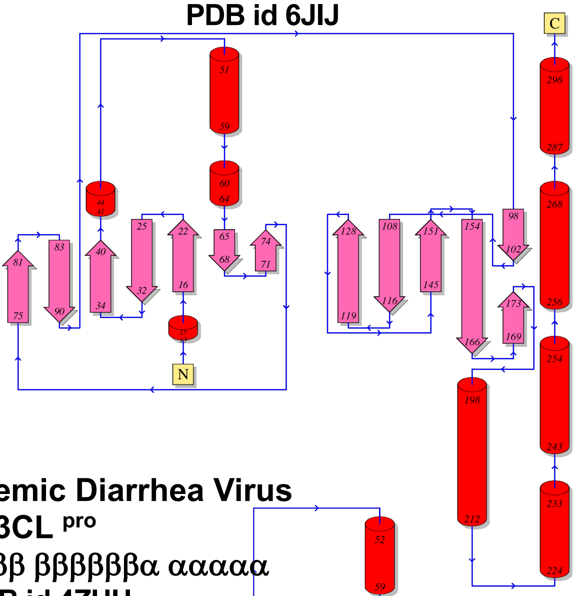

SARS CoV2 3CL pro
αβββαααββββ ββββββ ααααα
PDB id 6Y2G

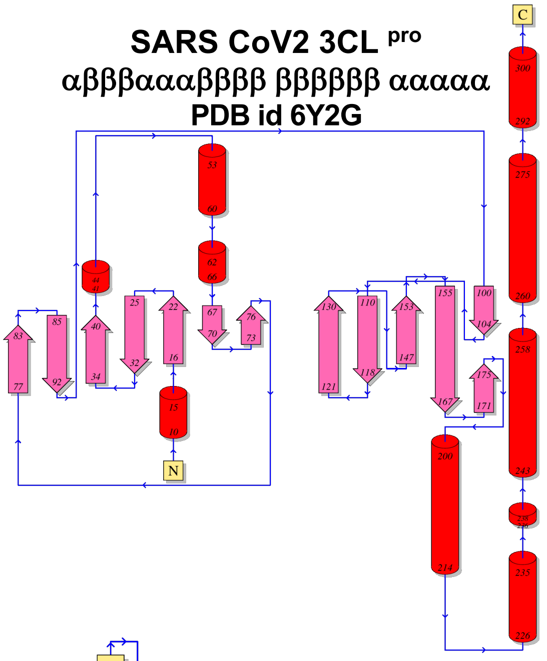

Porcine Epidemic Diarrhea Virus 3CL pro
αβββαααββββ ββββββα ααααα
PDB id 4ZUH

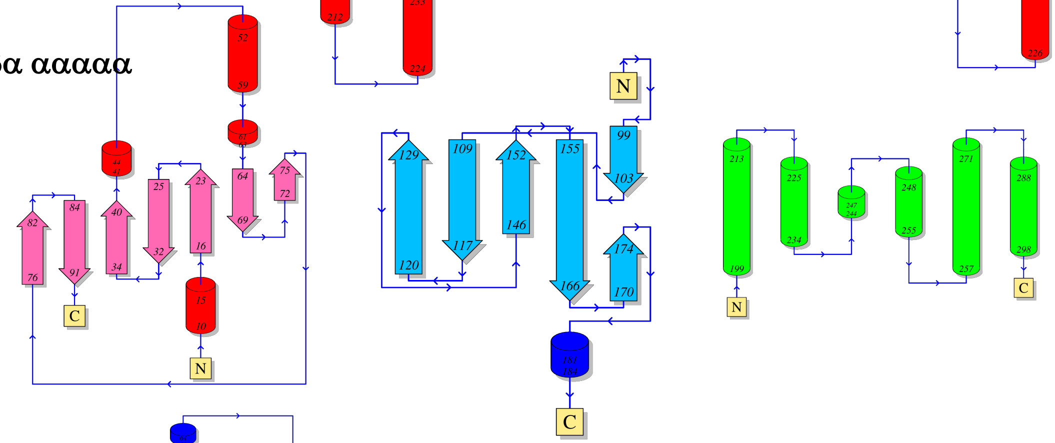

MERS 3CL pro
αβββααααββββ ββββββα ααααα
PDB id 4WME

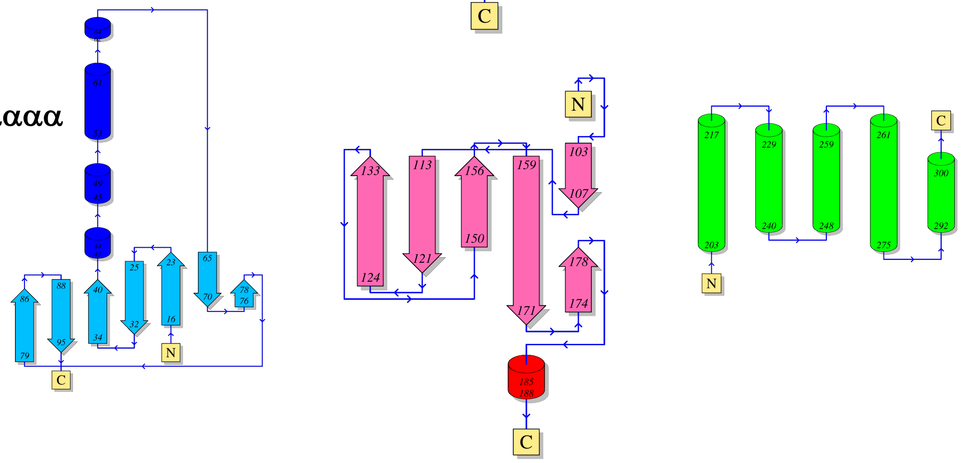

Infectious Bronchitis Virus 3CL pro
αβββααααββββ ββββββα ααααα
PDB id 2Q6F

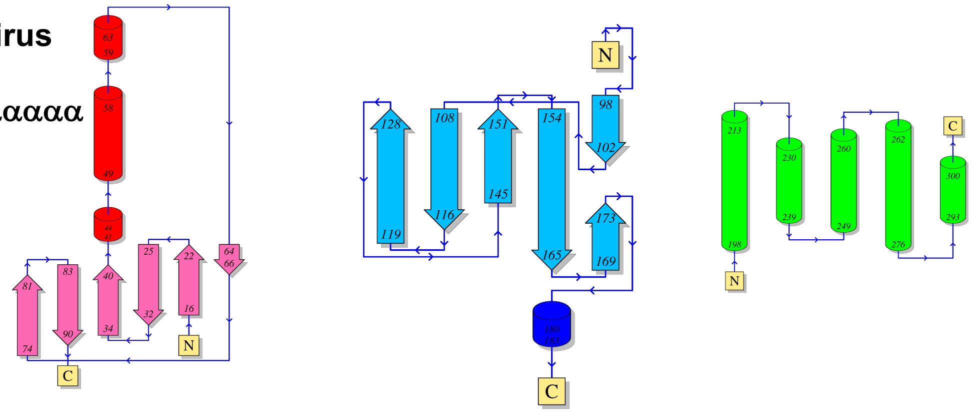

Figure S6: Comparison of topological representation of secondary structural elements of viral proteases of *Flaviridae* Family. α-helices are represented as cylinders and β- sheets are represented as arrows. These topological diagrams were obtained from *PDBsum* (Laskowski et al, 2018) with the indicated PDB id for each protease.

Dengue Virus NS2B/NS3 protease
ββββαββββ ββββββββββ
PDB id 3L6P

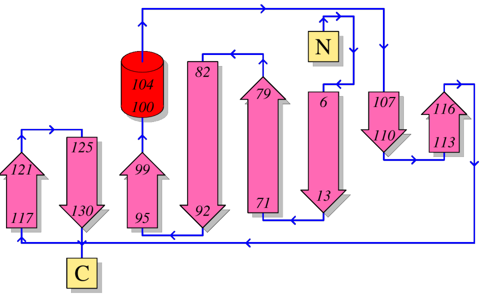

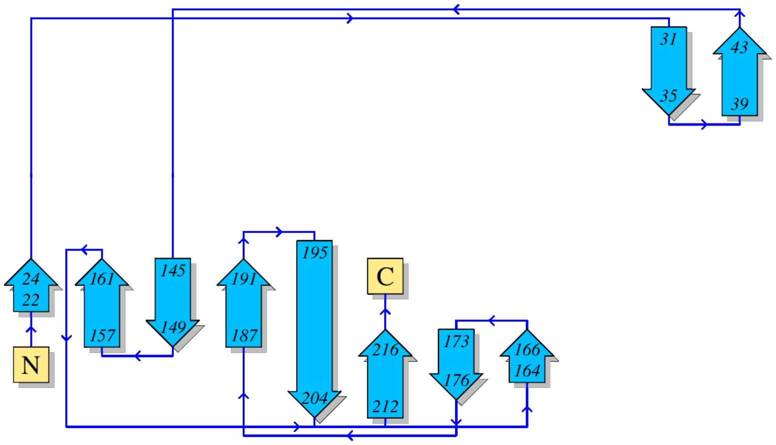

Zika Virus NS2B/NS3 protease
βββββββαβββββββββββ
PDB id 5LC0

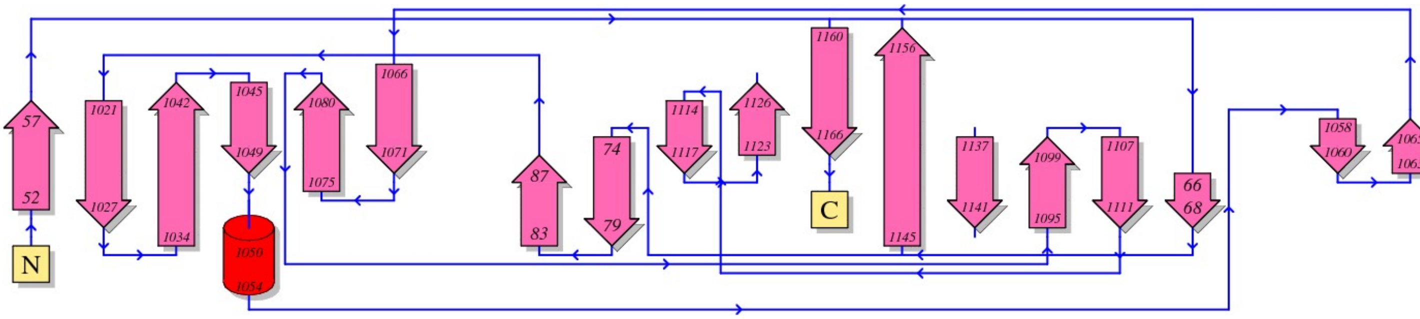

West Nile Virus NS2B/NS3 protease
βββαββββ ββββαβββ
PDB id 2GGV

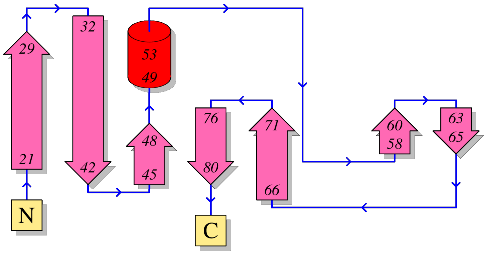

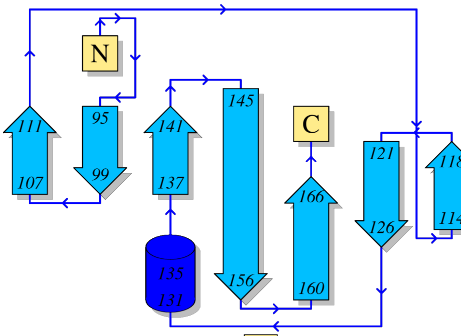

Hepatitis C Virus NS3/NS4A protease
βββαββαβ βββαβββα
PDB id 2P59

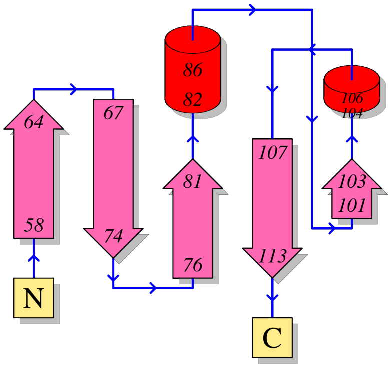

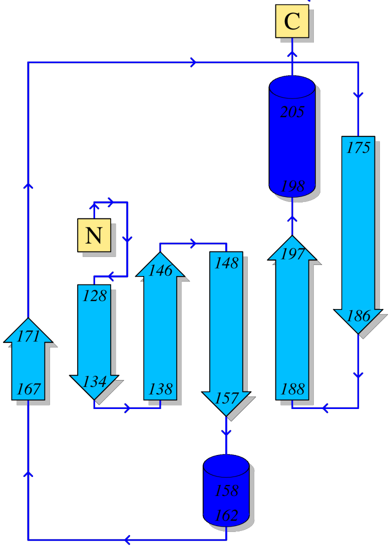

Figure S7: Docking of HCV protease NS3/4A inhibitor drugs to SARS CoV2 3CL^{pro} . Top panels - Molecular structures of two HCV protease inhibitor drugs. Middle panels – Lowest energy *AutoDock* pose of these HCV protease inhibitors (orange sticks) in the SARS CoV2 3CL^{pro} active site, Bottom panels – Details of atomic interactions in the lowest energy *AutoDock* poses of these HCV protease inhibitors. Hydrogen bonds and hydrophobic interactions between the drug and the enzyme are shown with yellow solid lines and black dashed lines, respectively. Sidechains of catalytic residues His41 and Cys145 are labeled, along with other protein residues that form key interactions with these drugs.

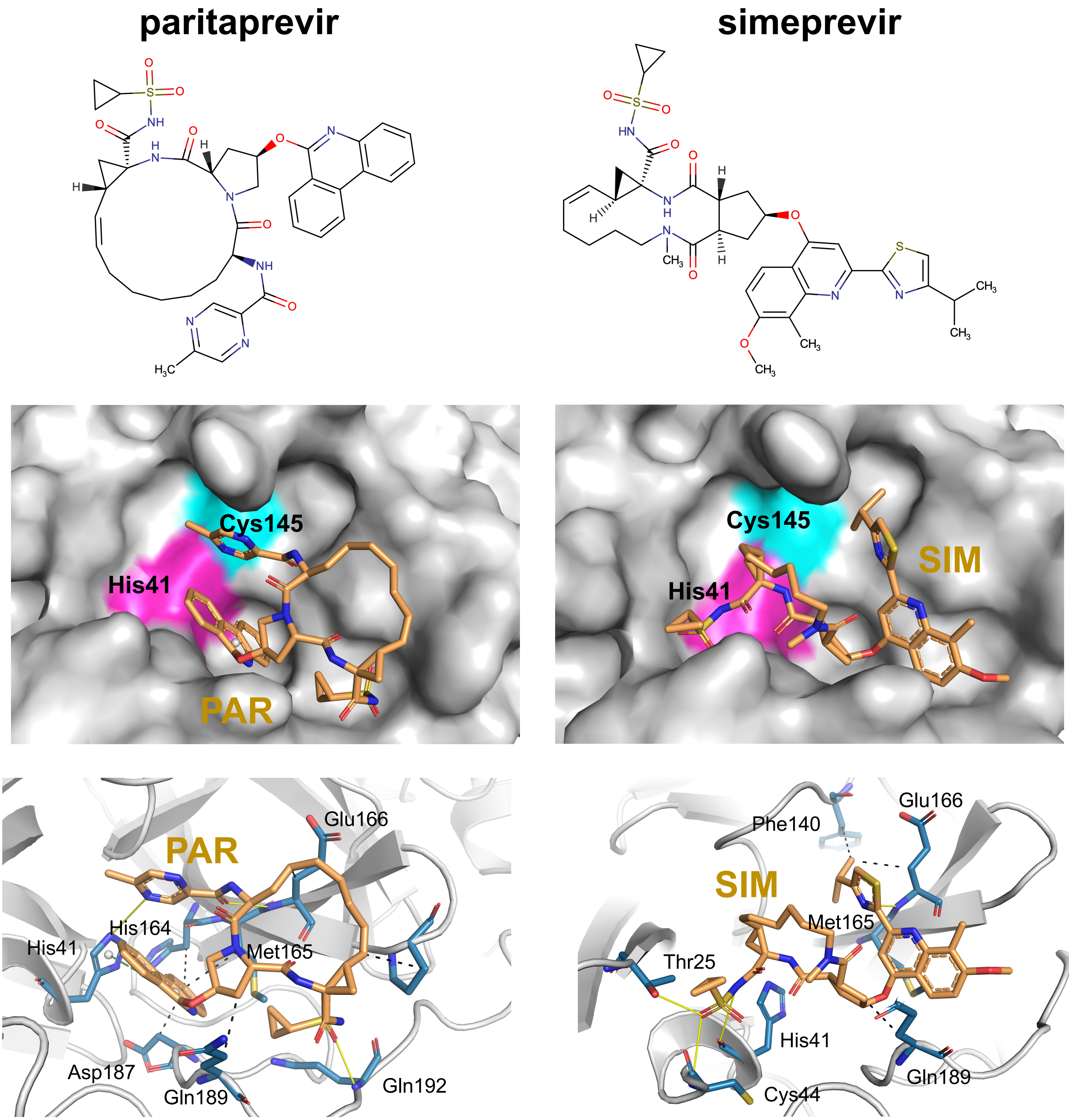

Figure S8: Docking of HCV protease NS3/4A inhibitor drugs to SARS CoV2 3CL^{pro} . Top panels - Molecular structures of two HCV protease inhibitor drugs. Middle panels – Lowest energy *AutoDock* pose of these HCV protease inhibitors (orange sticks) in the SARS CoV2 3CL^{pro} active site, Bottom panels – Details of atomic interactions in the lowest energy *AutoDock* poses of these HCV protease inhibitors. Hydrogen bonds and hydrophobic interactions between the drug and the enzyme are shown with yellow solid lines and black dashed lines, respectively. Sidechains of catalytic residues His41 and Cys145 are labeled, along with other protein residues that form key interactions with these drugs.

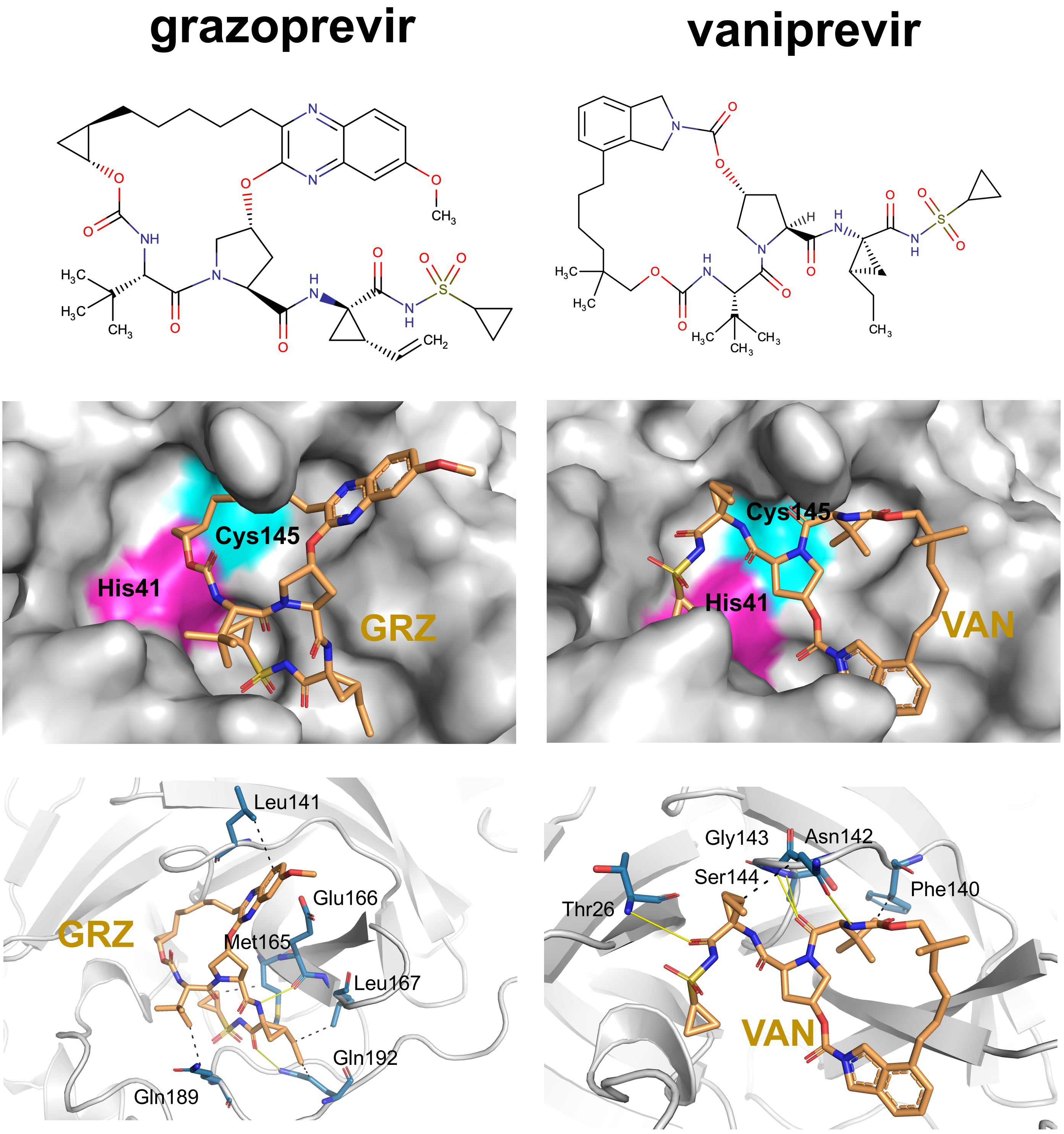

Figure S9: Chemical structures of 20 top-scoring 3C-like protease inhibitors listed in Table 3.

Nelfinavir
(1UN)

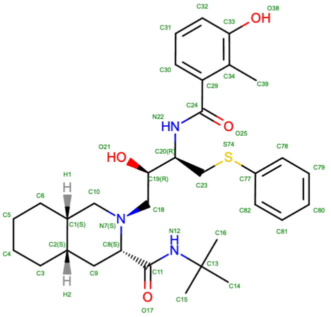

Triazol-based
macrocyclic inhibitor
(5LJ)

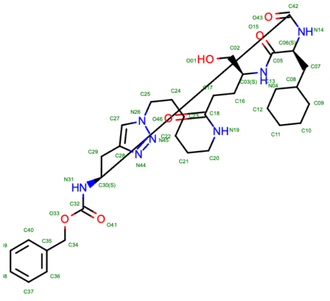

Triazol-based
macrocyclic inhibitor
(DW4)

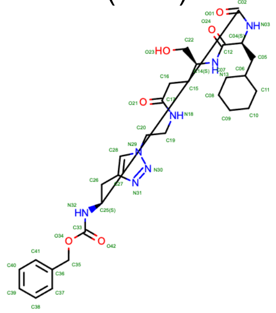

α,β -unsaturated
ethyl ester inhibitor
(G84)

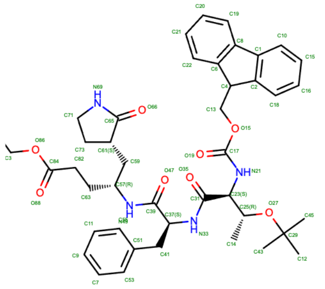

Compound 15
(DE6)

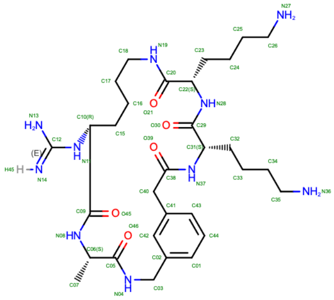

Analog of Rupintrivir
(γ-phenyl substitution)
(6OY)

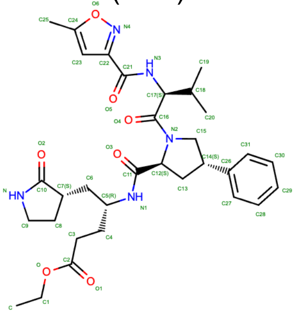

Compound 4
(DUU)

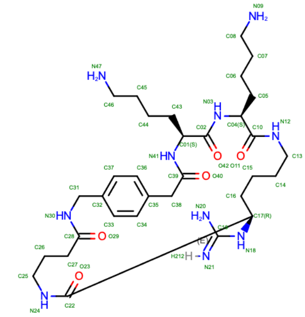

AG7404
(XNV)

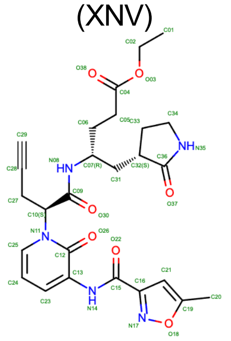

Dipeptidyl inhibitor
(N40)

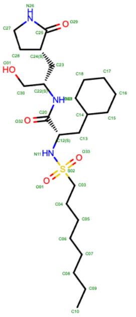

Compound 8
(DT0)

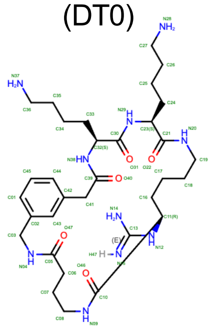

Bromocriptine
(08Y)

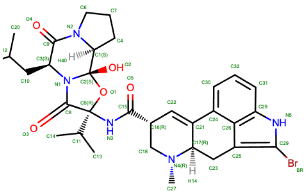

Rupintrivir
(AG7)

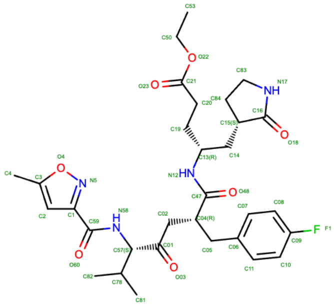

PRD_002347
(FHR)

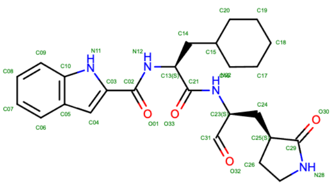

Triazol-based
macrocyclic inhibitor
(DW7)

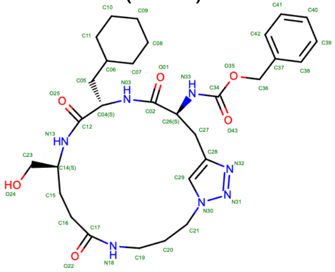

α,β -unsaturated
ethyl ester inhibitor
(G83)

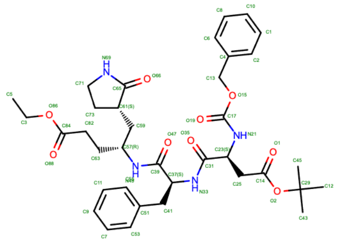

PRD_002214
Peptide-like inhibitor N3
(JIJ)

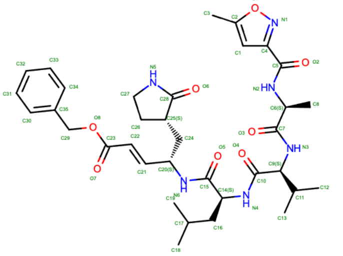

Compound 9
(DE0)

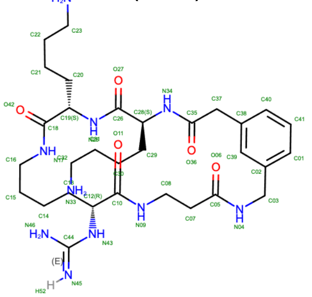

Novobiocin
(NOV)

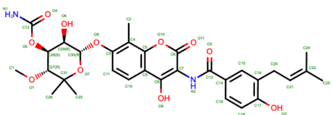

α,β -unsaturated
ethyl ester inhibitor
(G85)

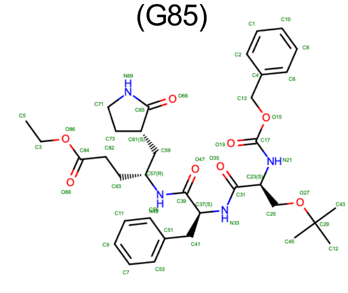

Compound 16
(DV0)

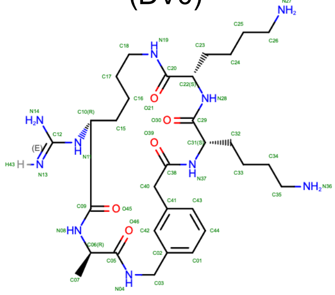

Supplement: Supplementary file 1 [file DataSheet1.PDF]
